# Differential methylation of the *TRPA1* promoter in pain sensitivity

**DOI:** 10.1038/ncomms3978

**Published:** 2014-02-04

**Authors:** J.T. Bell, A.K. Loomis, L.M. Butcher, F. Gao, B. Zhang, C.L. Hyde, J. Sun, H. Wu, K. Ward, J. Harris, S. Scollen, M.N. Davies, L.C. Schalkwyk, J. Mill, The MuTHER Consortium, Kourosh R. Ahmadi, Chrysanthi Ainali, Amy Barrett, Veronique Bataille, Jordana T. Bell, Alfonso Buil, Panos Deloukas, Emmanoil T. Dermitzakis, Antigone S. Dimas, Richard Durbin, Daniel Glass, Elin Grundberg, Neelam Hassanali, Asa K. Hedman, Catherine Ingle, David Knowles, Maria Krestyaninova, Cecilia M. Lindgren, Christopher E. Lowe, Mark I. McCarthy, Eshwar Meduri, Paola di Meglio, Josine L. Min, Stephen B. Montgomery, Frank O. Nestle, Alexandra C. Nica, James Nisbet, Stephen O’Rahilly, Leopold Parts, Simon Potter, Magdalena Sekowska, So-Youn Shin, Kerrin S. Small, Nicole Soranzo, Tim D. Spector, Gabriela Surdulescu, Mary E. Travers, Loukia Tsaprouni, Sophia Tsoka, Alicja Wilk, Tsun-Po Yang, Krina T. Zondervan, F.M.K. Williams, N. Li, P. Deloukas, S. Beck, S.B. McMahon, J. Wang, S.L. John, T.D. Spector

**Affiliations:** 1 Department of Twin Research and Genetics Epidemiology, Kings College London, London SE1 7EH, UK; 2 Wellcome Trust Centre for Human Genetics, University of Oxford, Oxford OX3 7BN, UK; 3 Pfizer Research Laboratories, Groton, Connecticut 06340, USA; 4 Medical Genomics, UCL Cancer Institute, University College London, London WC1E 6BT, UK; 5 BGI-Shenzhen, Shenzhen 518083, China; 6 Pfizer Limited, Neusentis, The Portway Building, Granta Park, Cambridge CB21 6GS, UK; 7 Institute of Psychiatry, King’s College London, London SE5 8AF, UK; 8 University of Exeter Medical School, University of Exeter, Exeter EX1 2LU, UK; 9 William Harvey Research Institute, Barts and The London School of Medicine and Dentistry, Queen Mary University of London, EC1M 6BQ, UK; 10 Wellcome Trust Sanger Institute, Hinxton CB10 1SA, UK; 11 Princess Al-Jawhara Al-Brahim Centre of Excellence in Research of Hereditary Disorders (PACER-HD), King Abdulaziz University, Jeddah 21589, Saudi Arabia; 12 Wolfson Centre for Age-Related Diseases, King’s College London, London SE1 1UL, UK; 13 Department of Biology, University of Copenhagen, Ole Maaløes Vej 5, 2200 Copenhagen, Denmark; 14 Macau University of Science and Technology, Avenida Wai long, Taipa, Macau 999078, China; 15 Department of Informatics, School of Natural and Mathematical Sciences, King’s College London, London, WC2R 2LS, UK; 16 Oxford Centre for Diabetes, Endocrinology & Metabolism, University of Oxford, Churchill Hospital, Oxford, OX3 7LJ, UK; 17 Department of Genetic Medicine and Development, University of Geneva Medical School, Geneva, 1211, Switzerland; 18 Biomedical Sciences Research Center Al. Fleming, 16672 Vari, Greece; 19 St. John’s Institute of Dermatology, King’s College London, London, SE1 9RT, UK; 20 University of Cambridge, Cambridge, CB3 0WA, UK; 21 European Bioinformatics Institute, Hinxton, CB10 1SD, UK; 22 University of Cambridge Metabolic Research Labs, Institute of Metabolic Science, Addenbrooke’s Hospital, Cambridge, CB2 0QQ, UK; 23 Cambridge NIHR Biomedical Research Centre, Addenbrooke’s Hospital, Cambridge, CB2 0QQ, UK; 24 Oxford NIHR Biomedical Research Centre, Churchill Hospital, Oxford, OX3 7LE, UK; 25 A full list of the MuTHER consortium and their affiliations appears at the end of the paper.

## Abstract

Chronic pain is a global public health problem, but the underlying molecular mechanisms are not fully understood. Here we examine genome-wide DNA methylation, first in 50 identical twins discordant for heat pain sensitivity and then in 50 further unrelated individuals. Whole-blood DNA methylation was characterized at 5.2 million loci by MeDIP sequencing and assessed longitudinally to identify differentially methylated regions associated with high or low pain sensitivity (pain DMRs). Nine meta-analysis pain DMRs show robust evidence for association (false discovery rate 5%) with the strongest signal in the pain gene *TRPA1* (*P*=1.2 × 10^−13^). Several pain DMRs show longitudinal stability consistent with susceptibility effects, have similar methylation levels in the brain and altered expression in the skin. Our approach identifies epigenetic changes in both novel and established candidate genes that provide molecular insights into pain and may generalize to other complex traits.

## Abstract (web summary)

Genetically identical twins provide a valuable resource to identify epigenetic factors associated with complex traits. Here the authors adopt this approach and find that differential methylation of the pain gene *TRPA1* is associated with pain sensitivity in humans.

## Graphical abstract

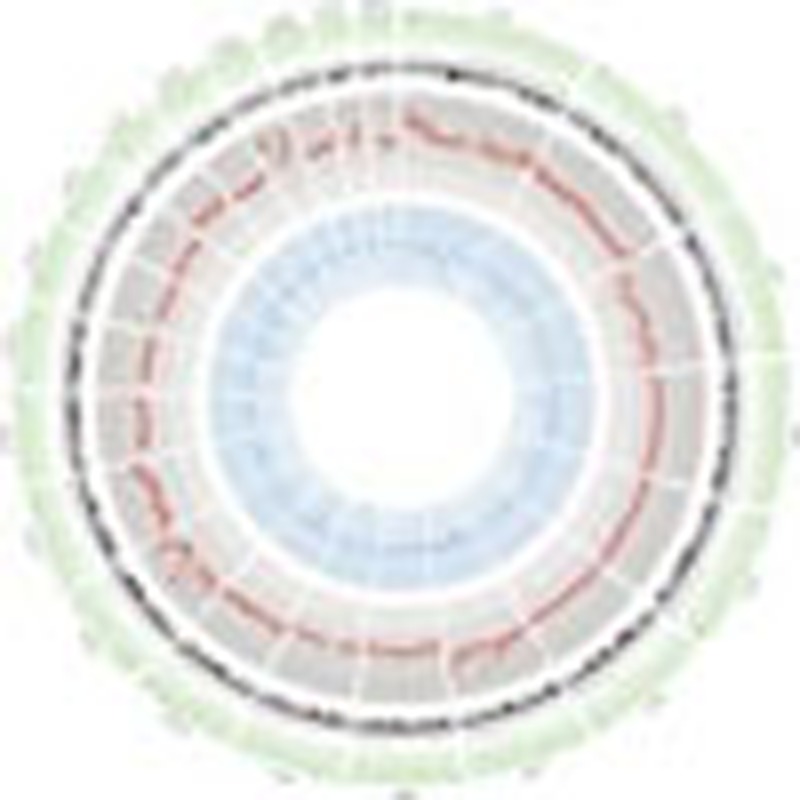

Acute and chronic pain have an impact on 20% of the population worldwide and represent a significant therapeutic and economic challenge^1^,^2^. Sensitivity to pain is a complex phenotype, which reflects the contribution of multiple biological, psychological and environmental risk factors. There is substantial variation in individual pain sensitivity^3^, only about half of which is explained by genetic effects, based on evidence from animal studies^4^,^5^, twin-based heritability estimates^6^,^7^ and genetic association analyses^8^. Multiple experimental approaches have been used to study pain mechanisms^9^,^10^. Chronic pain is known to be underpinned by several biological processes that together serve to amplify pain-related signals. The most well characterized of these are sensitization of the nociceptive nerve fibres (that innervate peripheral tissues), sensitization of the spinal circuits relaying signals associated with tissue damage and strong cortical modulation reflecting expectation, mood and past history. Recent evidence points to the involvement of epigenetic mechanisms across these molecular systems, both in the development and maintenance of pain states^11^,^12^.

DNA methylation is an essential epigenetic mechanism, which in mammals occurs at cytosine residues, predominantly in the context of CpG dinucleotides. DNA methylation is relevant for gene expression regulation, genomic imprinting, development and genomic stability. Emerging epigenome-wide association studies (EWAS) in humans suggest that epigenetic modifications may play a pivotal role in complex traits^13^. Recent EWAS of DNA methylation differences in disease-discordant monozygotic (MZ) twins have identified potentially differentially methylated regions (DMRs) in several complex diseases, ranging from diabetes to schizophrenia^14^,^15^,^16^. Epigenetic analyses of discordant twins can provide a powerful study design, but the power of this approach has not yet been tested in large samples at high resolution. Here we performed detailed genome-wide analyses of DNA methylation and identified modest but consistent and significant DNA methylation changes associated with pain sensitivity, with implications for epigenetic studies of other complex traits.

## Results

### Epigenome-wide association study design

We obtained DNA methylation sequencing profiles in a discovery sample of 25 MZ twin pairs (50 MZ twins) who were discordant for heat pain sensitivity (heat pain tolerance >2 °C between twins) determined experimentally using quantitative sensory testing (QST) (Supplementary Fig. S1). QST is commonly used for the assessment of pain sensitivity and the heat pain tolerance response (measured using the heat pain suprathreshold (HPST)) has been shown to be a clinically relevant QST measure^17^. We then obtained DNA methylation levels in a second independent sample of 50 unrelated individuals who did not overlap with the individuals in the discovery sample of MZ twins (Supplementary Fig. S1). Whole-blood DNA methylation patterns were assayed using methylated DNA immunoprecipitation followed by deep sequencing (MeDIP-seq), performed separately in the MZ twin sample and in the sample of unrelated individuals, resulting in an average of 50 million paired-end reads per individual in the MZ twin sample and an average of 25 million single-end reads per individual in the unrelated sample. We quantified MeDIP-seq reads to obtain a score of DNA methylation in overlapping 1 kb bins across the genome (Supplementary Figs S2 and S3). To identify DMRs associated with heat pain sensitivity (pain DMRs) we performed two separate pain-sensitivity EWAS on the two cohorts. To combine the results from the MZ twin and unrelated individual cohorts, we pursued a meta-analysis (MA) approach. MA is a powerful tool for aggregating information from multiple independent studies^18^ and is routinely used for pooling results from genome-wide association studies^19^, where it has successfully identified many disease-associated genetic variants with small effect sizes, not revealed in the individual studies^20^,^21^,^22^. We thereby obtained MA pain DMRs (MAP-DMRs) that were strongly supported by both cohorts and further investigated.

### Genome-wide patterns of DNA methylation in MZ twins

**Figure 1:**
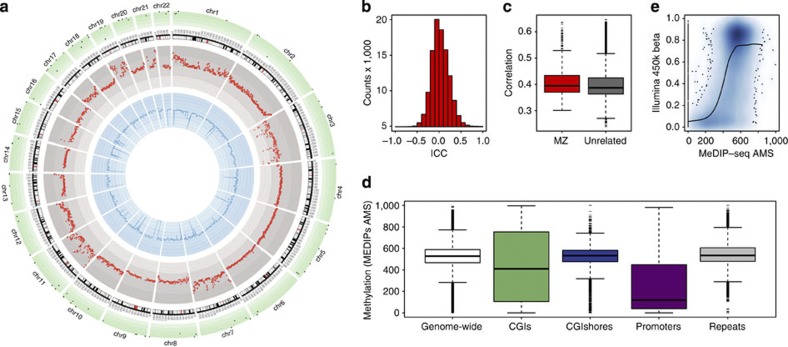
DNA methylation in MZ twins. CpG-density-weighted DNA methylation levels in 25 MZ discovery twin pairs showing. (**a**) DNA methylation (blue inner circle), within-pair MZ intraclass correlation (ICC) (red middle circle) and ASM levels (green outer circle). Methylation levels using running medians in 1-Mb windows are shown from low (AMS=0, light blue) to high (AMS=1,000, blue), and MZ ICCs are plotted from −0.25 (inner red circle radius) to 0.3 (outer red circle radius). ASM green circle represents the proportion of individuals that show evidence for ASM from 0.8 (light green) to 1 (green) at the 106 SNPs. (**b**) Genome-wide distribution of MZ ICCs and (**c**) correlations within MZ twins and unrelated pairs in the discovery MZ twin cohort (box shows the 25 and 75% quantiles and whiskers extend to 1.5 times the inner quartile range (IQR)). (**d**) DNA methylation levels across genomic annotations. (**e**) Within-individual MeDIP-seq and Illumina-450k DNA methylation comparison, showing the density of points in blue.

The DNA methylation sequencing data allowed us to assess at high resolution the distribution of DNA methylation patterns across the genome. We observed greater correlation in DNA methylation profiles within MZ pairs compared with pairs of unrelated individuals, as expected and consistent with previous studies^23^ (Fig. 1a–c). At a few genomic regions, the correlation pattern was reversed (Fig. 1c), potentially highlighting regions of high DNA methylation turnover. However, at the majority of loci across the genome DNA methylation patterns were positively correlated within MZ twins. The intraclass correlation coefficient within MZ twins across the genome was estimated at 0.064 per 1 kb bin (Fig. 1b), but the estimates showed variability across different regions and at different resolutions (Fig. 1a). We assessed evidence for allele-specific methylation (ASM) in the discovery set of 50 MZ twins and observed ASM (Methods) at 37.8% of tested autosomal heterozygous variants within an individual (considering only variants that were heterozygous in at least 50% of the sample). Overall, 106 heterozygous variants had evidence for ASM in at least 50% of the sample and >99% of these variants showed concordance for ASM of the same allele (or ASM skew) within MZ twin pairs (Fig. 1a). As expected, DNA methylation levels were also strongly correlated with genomic location and with previously annotated epigenetic variants and regulatory regions (Fig. 1d and Supplementary Fig. S4), where CpG islands and promoters had significantly reduced levels of DNA methylation.

### Pain-sensitivity epigenome-wide analyses

We tested for association between DNA methylation variation and pain sensitivity to identify DMRs related to pain (pain DMRs). We first performed pain-sensitivity EWAS in the discovery (MZ twin) and second (unrelated individuals) cohorts separately. In the discovery sample of 50 twins, we compared DNA methylation scores in 1 kb bins across the genome to pain scores for each individual, using a linear mixed-effects regression (LMER) model controlling for family structure (Supplementary Fig. S5 and Supplementary Table S1). At a permutation-based false discovery rate (FDR) of 5% (nominal LMER *P*=8.4 × 10^−9^), there was one significant pain DMR in an intergenic region on chromosome 3 (LMER *P*=8.4 × 10^−9^). Among the remaining top-ranked pain DMRs was *MVP* (ranked fourth; LMER *P*=7.9 × 10^−8^), which is involved in the interferon-γ signalling pathway^24^, previously implicated in neuropathic pain^25^. We also estimated pain DMRs within specific genomic annotation categories (CpG islands, CpG-island shores, and promoters) and found that the top-ranked regions included the pain gene *TRPA1* (ranked fourth, CpG-island shores, LMER *P*=3.9 × 10^−5^) and other potential pain candidates (*BAIAP2* (ranked first, CpG islands, LMER *P*=4.9 × 10^−5^) and *GRIN1* (ranked ninth, CpG islands, LMER *P*=2.9 × 10^−4^); Supplementary Methods). Among the 100 top-ranked regions in the CpG-island shore analysis were two additional known pain genes: the heat pain sensitivity gene *TRPV1* (ranked 31st, CpG-island shores, LMER *P*=2.3 × 10^−4^) and *TRPV3* (ranked 87th, CpG-island shores, LMER *P*=6.6 × 10^−4^), both within the same family of transient receptor potential channels as *TRPA1*. We then performed a pain-sensitivity EWAS in the second sample of 50 unrelated individuals and identified 22 pain DMRs at a permutation-based FDR of 5% (LMER *P*=2.5 × 10^−7^; Supplementary Fig. S6 and Supplementary Table S2). The most associated pain DMR in the unrelated samples was in *ANK3* (LMER *P*=1.9 × 10^−8^), which is required for normal clustering of voltage-gated sodium channels and normal action potential firing in neurons^26^, and is a susceptibility locus for bipolar disorder^27^. Pain DMRs in the second cohort also included *DCLK1* (ranked ninth, LMER *P*=1.2 × 10^−7^), which is a candidate gene for inflammatory nociception in mice^28^. To explore potential structure and confounders in the second cohort of unrelated individuals, we tested whether including multiple principal components altered the EWAS findings. We found that including the first up to third principal components as covariates did not change the top 22 association signals (Supplementary Fig. S6 and Supplementary Table S3).

We explored the sensitivity of EWAS results to potential confounders. We tested the influence of methylation normalization and the inclusion of covariates such as age, but did not find significant alterations in the top-ranked results (Supplementary Methods). To assess the sensitivity of pain DMRs to methylation quantification of MeDIP-seq data, we also performed analyses controlling for CpG density^29^ and MeDIP-seq fragment size in the discovery sample (Supplementary Figs S7 and S8), and found that the majority of top-ranked pain DMRs remained nominally significant, although their relative rank could change. For example, the *TRPA1* pain DMR (originally ranked 50th, LMER *P*=2.6 × 10^−6^) was nominally significant when controlling for MeDIP-seq fragment size (ranked 49th, LMER *P*=2.6 × 10^−6^) and CpG density (ranked 36,921st (in top 0.07%), LMER *P*=7.8 × 10^−3^).

To validate DNA methylation signals based on high-resolution MeDIP-seq, we obtained DNA methylation profiles measured on the Illumina Infinium 450k BeadChip assay^30^ in 44 individuals from the discovery sample. We compared genome-wide DNA methylation obtained from MeDIP-seq and 450k platforms in 1-kb regions genome wide and observed positive correlations (mean linear correlation=0.62) within individuals at 422,158 one-kilobase regions genome wide (Fig. 1e). The comparisons were performed using mean level of DNA methylation in 1-kb regions where data were available on both platforms (which included only 10% of regions assayed by MeDIP-seq and over 99.9% of CpG sites on the Illumina 450k array). We also performed EWAS for pain sensitivity using the Illumina 450k DNA methylation data, but the results did not surpass permutation-based FDR 5% (Supplementary Fig. S9). However, most MeDIP-seq pain DMRs were not represented on the 450k array and only two of the top ten discovery pain DMRs overlapped a 450k CpG site.

### Pain sensitivity MA

**Figure 2:**
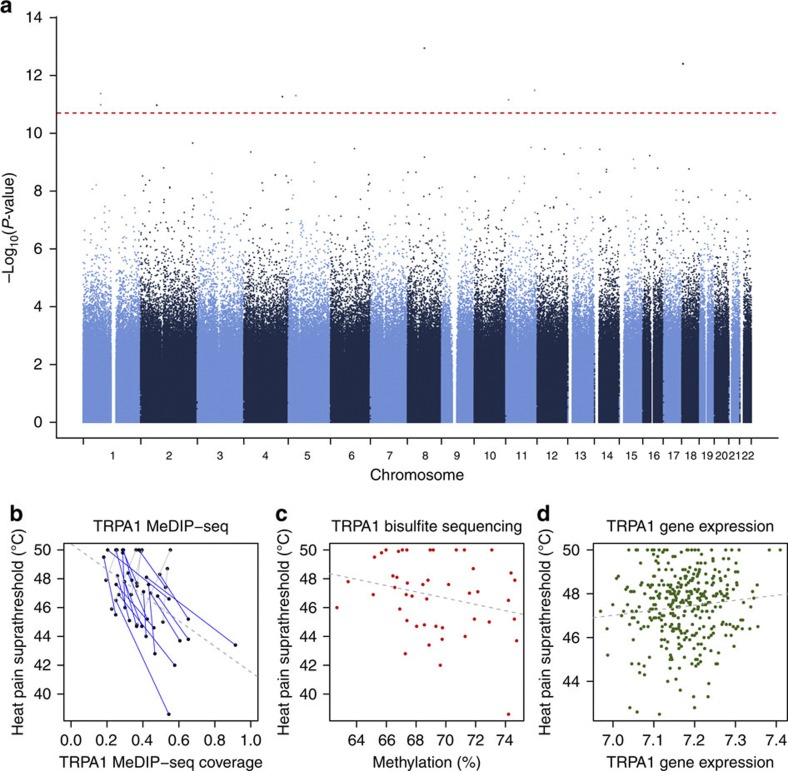
Meta-analysis pain EWAS results. (**a**) EWAS results and FDR 5% threshold (red line). (**b**,**c**) Differential methylation in *TRPA1* in pain sensitivity, showing (**b**) MeDIP-seq hypermethylation (blue) effects in the discovery set (co-twins linked by lines). (**c**) Pain DMR validates in the bisulphite sequencing data from CpG site at chr8:73151235, bp (pain-DMR rank correlation=0.22 and association taking into account twin structure, *P*=0.03). (**d**) Gene expression increases (LMER *P*=0.03) with higher pain thresholds in 341 twins.

We combined the EWAS results from the two cohorts using a fixed-effect epigenome-wide association MA in the combined sample of 100 individuals (Fig. 2a). There were nine MAP-DMRs with compelling evidence for association at a permutation-based MA threshold of FDR=5% (MA *P*=2.0 × 10^−11^), excluding results with evidence for heterogeneity. The nine MAP-DMRs mapped to eight unique regions in the *TRPA1* promoter (MA *P*=1.2 × 10^−13^), in an intergenic region on chromosome 18 (MA *P*=4.1 × 10^−13^), 17 kb from the *OR8B8* transcription start site (TSS) (MA *P*=3.4 × 10^−12^), 47–48 kb from the *ST6GALNAC3* TSS (MA *P*=4.4 × 10^−12^), in an intron of *MTMR12* (MA *P*=5.1 × 10^−12^), in an intergenic region on chromosome 4 (MA *P*=5.6 × 10^−12^), 33 kb from the *MICAL2* TSS (MA *P*=7.2 × 10^−12^) and 18 kb from the *NFU1* TSS (MA *P*=1.2 × 10^−11^; Table 1). In the majority of regions, DNA methylation levels were greater in individuals with low pain scores (hypermethylated MAP-DMRs). We assessed repeat content at MAP-DMRs (Table 1) and identified three MAP-DMRs (in *MTMR12*, *NFU1* and on chromosome 4) in high repeat content regions (>50% repeats). In addition, the second and third top-ranked MAP-DMRs (on chromosome 18 and in *OR8B8*) had low repeat content (<30%), but the DMR boundaries and surrounding genomic regions had high repeat content, which may influence DNA methylation quantifications. We also repeated the MA using the second sample EWAS corrected for methylation principal components and observed that the nine MAP-DMRs remained top ranked (Supplementary Table S4).

The strongest association signal was in the promoter of the ion channel gene *TRPA1* (Fig. 2b,c). *TRPA1* is a ligand-gated ion channel selectively expressed in peripheral nociceptors^31^,^32^ but also present in some non-neuronal cells, including keratinocytes^33^. The *TRPA1* promoter pain DMR in our data was hypermethylated in individuals with lower pain thresholds, and promoter DNA methylation can downregulate gene expression^34^. To assess whether promoter DNA methylation differences in *TRPA1* reflected functional consequences in pain, we obtained gene expression data from skin biopsies in 341 unselected normal female twins^35^. We observed a modest nominally significant (LMER *P*=0.03) increase in *TRPA1* gene expression levels in individuals with higher pain thresholds (Fig. 2d), consistent with the hypermethylated pain DMR result. Although *TRPA1* has not been reported as a heat sensor in mammals, it can be regulated by and interact with the thermosensor *TRPV1* (ref. 36), which is also expressed in keratinocytes. Therefore, we considered evidence for differential methylation and expression at the three reported thermosensors *TRPV1*, *TRPV2* and *TRPV3*. We observed MAP-DMR effects in a *TRPV1* intron (chr17:3,425,000–3,426,000 bp, MA *β*=0.16, *P*=6.7 × 10^−4^), 7 kb from the *TRPV2* TSS (chr17:16,251,500–16,252,500 bp, MA *β*=0.12, *P*=3.2 × 10^−3^) and in a *TRPV3* intron (chr17:3,398,500–3,399,500 bp, MA *β*=0.13, *P*=8.2 × 10^−3^), as well as nominally increased expression of *TRPV2* in the skin (*P*=0.01), which was consistent with *TRPA1* expression results. We also examined evidence for differential methylation at 73 known pain candidates from animal models, molecular and genetic studies (Supplementary Fig. S10), and observed differential methylation in several pain candidate genes, including *KIAA0564* (MA *P*=2.9 × 10^−5^), *GRIA1* (MA *P*=2.9 × 10^−5^), *CACNA2D3* (MA *P*=8.4 × 10^−5^), *PRDM16* (MA *P*=9.4 × 10^−5^) and *P2RX3* (MA *P*=9.9 × 10^−5^).

Whole blood is a heterogeneous collection of cells and DNA methylation levels within particular genomic regions may in part reflect the cellular composition of the sample. To assess the extent of these associations in our data, we tested for association between white blood cell (WBC) subtype counts for lymphocytes, neutrophils, basophils and eosinophils, and DNA methylation levels at the nine FDR 5% MAP-DMRs in the discovery sample. We did not observe significant associations between DNA methylation at the nine MAP-DMRs with proportion of neutrophils, eosinophils or monocytes, but one of the MAP-DMRs, the intergenic MAP-DMR on chromosome 4 ranked 6th in Table 1, was associated with lymphocyte counts (LMER *P*=0.003). We conclude that variability in WBC subtypes does not have a major effect on the top-ranked pain sensitivity DMRs in our study.

### DMR validation by deep bisulphite pyrosequencing and Illumina 450k arrays

To validate DNA methylation levels obtained by MeDIP-seq at MAP-DMRs, we first compared MeDIP-seq with Illumina 450k DNA methylation estimates. However, only two MAP-DMRs (in *TRPA1* and *MICAL2*) had a single CpG site represented on the Illumina 450k platform within the 1-kb DMR. The *MICAL2* MAP-DMR did show a positive correlation in DNA methylation levels across the two platforms (rank correlation=0.25). To validate DNA methylation levels at the *TRPA1* MAP-DMR, we performed bisulphite pyrosequencing of 700 bp in the 1-kb MAP-DMR region. We validated DNA methylation levels at two CpG sites in a 200-bp region within the CpG-island shore (Fig. 2c and Supplementary Fig. S11). The CpG sites that validated the MeDIP-seq signal were not represented on the Illumina 450k array, but six other CpG sites on Illumina 450k that were within 500 bp of the *TRPA1* DMR boundary outside the DMR validated MeDIP-seq levels. We also considered validation by examining the direction of pain DMR effects in the EWAS results from the Illumina 450k platform at CpG sites allocated to the same gene as the MAP-DMRs. We found that four of the six genes (based on FDR 5% MAP-DMRs, that is, *TRPA1*, *ST6GALNAC3*, *MTMR12* and *MICAL2*) had consistent direction of pain-methylation associations in both MeDIP-seq and Illumina 450k platforms, and 52% of the 100 top-ranked gene-based MAP-DMRs validated using this approach.

### Exploring molecular mechanisms underlying DMRs

The pain DMRs identified in our study may contribute to pain sensitivity or arise as a consequence of pain. To tackle this question, we assessed whether DNA methylation levels at MAP-DMRs showed evidence of association with genetic variants in *cis*. We tested for methylation quantitative trait loci (meQTLs) in 47 unrelated individuals from the second cohort using common genetic variants (minor allele frequency ≥0.05) within 50 kb of the 9 MAP-DMRs. Six MAP-DMRs had evidence for *cis* meQTL effects, including *OR8B8* (meQTL *P*=5.8 × 10^−6^), both *ST6GALNAC3* DMRs (meQTL *P*=3.0 × 10^−5^) and modest effects at MAP-DMRs on chromosome 4 (meQTL *P*=0.01), in *TRPA1* (meQTL *P*=0.03) and on chromosome 18 (meQTL *P*=0.03; Supplementary Table S5). In comparison, 56% of the top 100 MAP-DMRs were associated with genetic variants in *cis* at nominal significance.

Differential methylation in pain sensitivity may also mediate or reflect environmental or unknown non-genetic risk factors in pain. Our data allowed us to explore such effects by comparing MZ twins, who are matched for most genetic variation, age, cohort and many early-life environmental effects. We compared within-pair differences in DNA methylation and heat pain tolerance in the discovery set, and observed an enrichment of negative correlations between methylation and heat pain tolerance twin differences, consistent with predominantly hypermethylated effects (Supplementary Fig. S12), including results at the FDR 5% MAP-DMRs (Table 1). Several of the 100 top-ranked MAP-DMRs (*MVP*, *DTNA*, *GLYATL1*, *FHIT*, *FBXO15* and *TMEM132D*), as well as known pain candidates such as *TRPM3*, *GRIA1* and *GRIA4* had strong evidence for association between within-pair differences in methylation and pain levels (*P*=1.0 × 10^−5^). We found no evidence of correlation between within-pair ASM skew and heat pain tolerance difference.

### Longitudinal DNA methylation profiles to assess DMR stability

**Figure 3:**
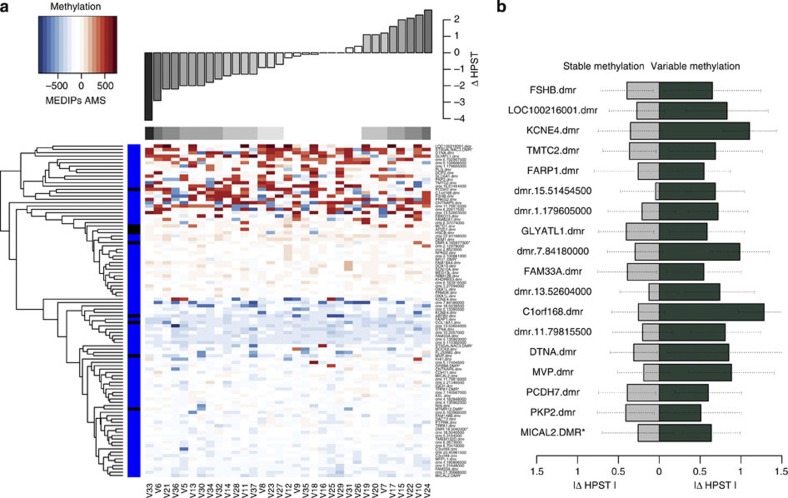
Longitudinal stability of pain DMRs. (**a**) DNA methylation differences (MEDIPS AMS) over 2–3 years in 33 discovery set individuals (columns), ordered by longitudinal differences in HPST scores (bar plot). Heatmap rows correspond to the 100 top-ranked MAP-DMRs, where the top 25% are highly variable and show loss (blue) and gain (red) of methylation over time. (**b**) Eighteen MAP-DMRs with greater HPST differences in individuals (*n*≥6) with variable methylation (change 30–100%) over time (green), compared with HPST differences in individuals with stable methylation (change 0–30%) over time (grey). Box shows the 25 and 75% quantiles and whiskers extend to 1.5 times the IQR.

To examine further the stability and potential contribution of epigenetic effects towards pain sensitivity, we obtained longitudinal MeDIP-seq and heat pain tolerance data for 33 individuals from the discovery set, sampled 2–3 years apart. We compared longitudinal differences in DNA methylation with longitudinal differences in HPST and found that the 100 top-ranked MAP-DMRs clustered in three groups (Fig. 3a). Although ~25% of regions had highly variable methylation over time, the majority of MAP-DMRs remained relatively stable and 15% showed a modest loss of methylation over time. Longitudinal DNA methylation patterns at the nine MAP-DMRs showed relatively stable methylation over time (Supplementary Fig. S13). At specific regions within the 100 top-ranked MAP-DMRs heat pain tolerance, changes were lower in individuals in whom DNA methylation remained stable over time and higher in individuals whose DNA methylation levels significantly changed with time, consistent with a model of epigenetic contribution to pain sensitivity. Altogether, there were at least 18 regions at which the longitudinal data were consistent with epigenetic susceptibility to pain, including MAP-DMRs in *MICAL2*, *MVP* and *KCNE4* (Fig. 3b and Supplementary Table S6).

### DNA methylation levels across tissues

Another factor in the current study was the relevance of DNA methylation changes observed in whole blood to heat pain tolerance. We therefore compared DNA methylation profiles of the most significant loci identified here (the MAP-DMRs) in blood and multiple brain regions from two female donors^37^, who were not part of the cohorts used in our EWAS results. We observed a significant positive correlation between mean level of methylation in blood and brain across the 100 top-ranked MAP-DMRs (linear correlation coefficient=0.33, *P*=8.5 × 10^−4^). Moreover, five of the nine significant MAP-DMRs had very similar methylation levels in the blood and brain (Supplementary Methods). There was significant variability in methylation levels across the multiple brain tissues at the top 100 MAP-DMRs, and the observed differences between blood and brain were as extreme as the methylation differences seen between cerebellum and other brain regions. Skin gene expression levels at MAP-DMR genes also showed negative correlations with DNA methylation, suggestive of potential functional effects. Together, these observations suggest that whole-blood DNA methylation levels at the MAP-DMRs are generally relevant to other biological tissues in the context of our study.

## Discussion

The aim of this study was to identify genomic regions that are differentially methylated in pain sensitivity. We hypothesized that DNA methylation changes may lead to or arise as a consequence of heat pain tolerance and thereby identify known and novel candidate genes involved in pain sensitivity. We characterized genome-wide DNA methylation profiles using MeDIP-seq in 100 individuals and compared these profiles with clinically validated measures of pain sensitivity. We used a two-stage EWAS design and combined our findings into an MA, which identified nine regions (MAP-DMRs) at a genome-wide significant FDR of 5%. DNA methylation levels at MAP-DMRs were validated using bisulphite-conversion methods. These regions also showed DNA methylation stability over time, consistent with susceptibility effects on measured pain sensitivity. Similar levels of DNA methylation were observed in blood and brain tissues at multiple pain DMRs, and negative correlations between gene expression and pain-DMR DNA methylation were observed at pain-DMR genes in the skin.

Overall, the strongest association MA signal was in the promoter of the ion channel gene *TRPA1*. *TRPA1* is a ligand-gated ion channel, which is directly gated by a range of irritants and noxious cold, and may also gate pain-related responses to a range of G-protein-coupled receptors. In some species, *TRPA1* acts as a heat sensor, for example, *TRPA1* is required for thermal nociception in *Drosophila*^38^,^39^,^40^, where a particular *TRPA1* isoform acts to reduce thermosensitivity^38^. Although *TRPA1* has not been reported as a heat sensor in mammals, it can be regulated by and interact with the thermosensor *TRPV1* (ref. 36). Both *TRPA1* and *TRPV1* are expressed in peripheral nociceptors^31^,^32^ but are also present in some non-neuronal cells, including keratinocytes^33^. Therefore, we considered evidence for differential methylation and expression at *TRPA1*, and the three thermosensors *TRPV1*, *TRPV2* and *TRPV3* in the skin. We observed nominally significant increased expression levels of *TRPA1* in the skin at higher pain thresholds, which is consistent with a downregulatory effect of DNA methylation in the *TRPA1* promoter pain DMR on *TRPA1* gene expression. We were not able to measure *TRPA1* expression in nociceptors. *TRPV1*, *TRPV2* and *TRPV3* had modest evidence for pain-DMR effects in whole-blood DNA methylation, suggesting an involvement of these thermosensors in differential pain sensitivity in our sample. In summary, our findings suggest the presence of a regulatory DNA methylation region in a CpG-island shore of the *TRPA1* promoter, which may have an impact on *TRPA1* gene expression and thermal sensitivity.

Our MA results included additional regions, which are compelling candidate genes for pain sensitivity. The fourth most associated MAP-DMR was 47 kb from the TSS of the *ST6GALNAC3* gene. *ST6GALNAC3* catalyses the transfer of sialic acids to carbohydrate groups on glycoproteins and glycolipids^41^. It was recently identified as a candidate gene for subcutaneous fat thickness in pigs^42^, suggesting that it may be a novel candidate for heat pain sensitivity in skin. The seventh most associated MAP-DMR was 33 kb from the TSS of *MICAL2*, which has a role in molecular processes highly relevant to pain sensitivity. *MICAL* proteins can control actin cytoskeleton dynamics and several aspects of neural development^43^, for example, in *Drosophila*, *MICAL* is required for axon guidance^44^ and is an essential regulator of synaptic structure along muscle fibres^44^, and contributes to dendritic pruning^45^. We also examined the 100 top-ranked MAP-DMRs (FDR=14%, *P*=3.0 × 10^−8^), which mapped to 53 genes, including *TRPA1* and *SCN10A* pain genes, and other genes linked to metal ion transport and binding (*MICAL2*, *KCNE4*, *CDH11*, *COL18A1*, *DTNA*, *FHIT*, *KEL*, *PCDH7*, *PLG* and *SLC8A1*), which are key processes in pain pathways, as well as cytoskeleton and cell adhesion, which may influence or mediate pain sensitivity. Gene ontology analyses^46^ highlighted significant term enrichment for several processes, including vasoconstriction.

The majority of epigenetic studies of human traits published to date have used whole blood, as it is often the only sample type that is available in many human cohorts. However, the suitability of blood as an appropriate tissue for epigenome-wide association studies of complex traits has been debated. Blood is a heterogeneous collection of cells, and DNA methylation levels may in part reflect cellular composition^47^. We assessed evidence for this using the top nine FDR 5% MAP-DMRs, but did not observe significant associations between DNA methylation levels and the proportion of lymphocytes, neutrophils, basophils and eosinophils. Therefore, variability in WBC subtypes did not appear to have a major effect on the top-ranked pain sensitivity DMRs in our study. Aside from blood cell-subtype heterogeneity, another issue is whether blood is the appropriate surrogate for the tissue manifesting the phenotype of interest^48^. In terms of pain sensitivity, we would ideally explore DNA methylation levels in nociceptors, peripheral nerve, dorsal root ganglion and the central nervous system, but these tissues were not available for the subjects in our study and, to our knowledge, such data have not been explored in humans in the context of pain. Therefore, we tested whether our peak DMRs had consistent levels of DNA methylation or gene expression in whole blood, multiple brain tissues and the skin. We concluded that many of the top-ranked DMRs showed consistent methylation or expression changes across blood and tissues relevant to pain, but further epigenetic studies in pain need to address this question genome wide across tissues.

We explored molecular mechanisms that may underlie DMRs for pain sensitivity. DNA meQTL analysis at the nine MAP-DMRs indicated the presence of strong genetic effects at MAP-DMRs in *OR8B8* and *ST6GALNAC3*, and modest genetic effects at *TRPA1* and two intergenic MAP-DMRs on chromosomes 4 and 18. Consistent with this, a second set of MAP-DMRs showed strong association in the MZ discordance analyses, which aim to identify MAP-DMRs that are not genetically determined. Together, this pair of analyses allow us to differentiate between regions at which genetic effects on pain sensitivity may be mediated through DNA methylation, and regions at which DMRs for pain sensitivity may have an environmental cause or may arise secondary to phenotype variation.

Longitudinal analyses at the 100 top-ranked MAP-DMRs were consistent with susceptibility DMR effects on pain sensitivity at multiple genomic regions, but it is difficult to establish susceptibility in the absence of a phenotype-onset time point. However, these results clearly highlighted the presence of both longitudinally stable and variable regions (Fig. 3a), of which the top nine MAP-DMRs were predominantly stable. As expected, the majority of MAP-DMRs with meQTLs showed evidence for longitudinal stability (Fig. 3), but a proportion of the top-ranked MAP-DMRs in the MZ discordance results was also longitudinally stable. We also attempted to identify MAP-DMRs with potential susceptibility effects, that is, where heat pain tolerance changes were lower in individuals in whom DNA methylation remained stable over time and higher in individuals in whom DNA methylation levels significantly changed with time, consistent with a model of epigenetic contribution to pain sensitivity.

In summary, we observed strong evidence for association between DNA methylation levels and pain-sensitivity scores in a data set of 100 individuals. DNA methylation levels at a subset of MAP-DMRs showed longitudinal correlation with heat pain tolerance stability, genetic associations in *cis*, similar patterns across blood and brain tissues and correlations with skin gene expression. Taken together, the results at these DMRs are consistent with an epigenetic influence on sensitivity to pain in normal human volunteers. Our findings confirm that susceptibility genes identified from epidemiological studies of complex traits show epigenetic modifications with probable functional alterations. Most of these regions identified were not represented on the Illumina 450k array, suggesting that a higher-density screening approach is essential to capture relevant methylation loci. These data have implications for other complex traits and suggest that epigenomic approaches may provide valuable insights into mechanisms linking genetic variation to complex phenotypes.

## Methods

### Subjects

All participants in the study provided written informed consent in accordance with the St Thomas’ Hospital Local Ethics Committee. Altogether, 100 volunteer, female MZ and dizygotic twins from the TwinsUK cohort^49^ were included in the study, including a discovery set of 50 MZ twins (25 MZ pairs) and a second independent set of 50 unrelated individuals. QST for heat pain tolerance was performed according to standard protocols (Supplementary Methods and included measures of the HPST. The discovery 25 MZ twin pairs (median age 62 years) were selected as the most discordant MZ twin pairs for HPST scores in the TwinsUK cohort at the time of the study (Supplementary Fig. S1). Discordance was defined as a difference of at least 2 °C in HPST scores, and one twin in each pair fell in the upper tail of the HPST distribution (low pain sensitivity). The second sample of 50 unrelated individuals (median age 64 years) was unselected and HPST scores were representative of the TwinsUK cohort HPST distribution (Supplementary Fig. S1). Whole blood was collected after QST and DNA was extracted using standard protocols. WBC subtype counts were obtained in 48 individuals from discovery sample using FACS of peripheral blood^50^. WBC subtype cell counts were calculated for four cell types: neutrophils, eosinophils, monocytes and lymphocytes.

### MeDIP sequencing

MeDIP-seq was performed and analysed separately in the MZ twin and unrelated samples. In the discovery MZ twin sample, 5 μg genomic DNA from each sample was sonicated on a Diagenode Bioruptor to produce a median fragment length of 180–230, and verified using a 2100 Bioanalyzer with DNA1000 chips (Agilent, Santa Clara, USA). Samples were prepared for next-generation sequencing by blunt ending, dA-tailing and then ligating paired-end adapters according to manufacturer’s reagents and protocols (Illumina) with purification (QIAGEN) between steps. Samples were quantified and size checked using a 2100 Bioanalyzer and DNA1000 chips (Agilent). One microgram of each adapter-ligated sample was spiked with ~5 × 10^−7^ pmoles methylated/unmethylated control DNA (see ref. 51 for details) and subjected to MeDIP (ref. 52) with 150 ng anti-5-methylcytidine using automation according to manufacturer’s reagents and protocols (Diagenode); 100 ng was reserved as input for MeDIP quality control (QC), which was conducted using quantitative PCR (qPCR) with primers designed to amplify methylated/unmethylated control DNA. MeDIP DNA was then purified (Zymo Research) and amplified by 18 cycles adapter-mediated PCR. PCR products were purified (QIAGEN) and run out on separate 2% low-melting point agarose gels. PCR fragments were excised and purified (QIAGEN). The resulting libraries (300–350 bp) were QC using an Agilent 2100 Bioanalyzer and DNA1000 chips and qPCR (see ref. 53). The samples were sequenced on Illumina GA2, using two lanes per sample, to produce on average 50 million paired-end reads of length 50 bp.

In the unrelated samples, 1.5 μg of original genomic DNA was fragmented to DNA smear between 200 and 500 bp using a Bioruptor NGS (Diagenode) sonication system. End repair, <A> base addition and adaptor ligation steps were performed using Illumina’s Paired-End DNA Sample Prep kit following the manufacturer’s instructions. Next, the methylated DNA fractions were immunoprecipitated using the Magnetic Methylated DNA Immunoprecipitation Kit (Diagenode, catalogue number: mc-magme-048) with small adjustments. Briefly, 1.5 μl of each two control templates (methylated and unmethylated DNA controls) with different sequences were mixed with adapter-ligated DNA and heat denatured (95 °C, 3 min) in a final reaction volume of 90 μl within the provided reagents of 24 μl MagBuffer A and 6 μl MagBuffer B. Aliquot (7.5 μl) of the denatured genomic DNA was saved as input and another 75 μl was immunoprecipitated with 5 μl of prepared anti-5mC antibody and 20 μl of prepared Magbeads. The DNA–antibody magbeads mixture was incubated overnight at 4 °C on a rotating wheel. The DNA–antibody magbeads mixture was then washed twice using each of 150 μl ice-cold MagWash Buffer-1 and washed once with 150 μl of ice-cold MagWash Buffer-2. For each buffer, the washing reaction was incubated for 4 min at 4 °C on a rotating wheel. The input DNA and immunoprecipitated products were then in parallel treated with protease K and purified with ZYMO DNA Clean & Concentrator-5 (ZYMO). The efficiency and sensitivity of immunoprecipitation reaction were detected by qPCR using the enriched DNA and the unbound input DNA. MeDIP-seq library was constructed by PCR amplification with the enriched DNA as template. PCR reaction was performed in 50 μl of reaction volume consisting of 20 μl MeDIP-enriched DNA, 5 μl of 2.5 mM dNTP, 2 μl primers, 5 μl of 10 × pfx amplification buffer, 2 μl of 50 mM MgSO_4_, 15.2 μl sterilized water and 0.8 μl of Platinum pfx DNA polymerase (Invitrogen). The programme of amplification was 94 °C 2 min, 10 cycles of 94 °C 15 s, 62 °C 30 s and 72 °C 30 s, then prolong with 10 min at 72 °C. The products could be kept at 12 °C. The PCR products were purified using Agencourt Ampure Beads (Beckman Coulter). After analysing by the Bioanalyzer analysis system (Agilent) and quantified by the real time PCR, the prepared library was sequenced using Illumina HiSeq2000 with SE50 read length.

### MeDIP-seq DNA methylation quantification

In the discovery MZ twin sample, we obtained on average 50 million paired-end reads of length 50 bp per individual. Alignment to the human genome (hg18) was performed using Maq^54^ (v0.7.1). QC of the aligned data took into account base quality scores (no exclusions), read mapping scores (threshold maq q>10), removal of duplicate paired-end (PE) reads, proper PE pairing criteria and insert-size distribution checks (see Supplementary Fig. S2 and Supplementary Methods). On average, 60% of reads passed alignment QC per individual. We used the post-QC PE reads to reconstruct MeDIP fragments per individual. We quantified coverage of MeDIP fragments per base pair across the genome and binned coverage into overlapping 1-kb bins (overlap of 500 bp). For each bin, we calculated a normalized methylation score, the relative methylation quantification (RMQ) score, which was the sum of the MeDIP-fragment coverage of each base pair in that bin, divided by the overall number of autosomal post-QC reads per individual. For pain-DMR analysis, we only considered bins with RMQ variance >0, where at least 10% of the sample had RMQ scores >0. There were 5,260,672 autosomal 1-kb bins used in the discovery pain-DMR analyses.

In the discovery sample, we also performed additional quantifications of MeDIP-seq data, taking into account variation in the size distribution of MeDIP fragments across individuals and variation in CpG density across genomic regions (Supplementary Methods and Supplementary Figs S6 and S7). For fragment analyses, we matched the distribution of MeDIP fragments across the 50 individuals to match a standardized distribution (Supplementary Methods), and in the analyses controlling for CpG density, we used MEDIPS^29^ to calculate absolute methylation scores (AMS). We used AMS to examine DNA methylation patterns across the genome (Fig. 1), and we used AMS and fragment-corrected scores to assess the reproducibility of pain DMRs to different MeDIP-seq quantifications (Supplementary Figs S7 and S8).

In the unrelated samples, we obtained on average 25 million single-end reads of length 50 bp per individual. Sequence alignment to the human genome (hg18) was performed in Bwa^55^ (v0.5.9; Supplementary Fig. S3). Data were subject to QC checks for base composition (no exclusions), read mapping quality (threshold bwa *q*>10) and removal of duplicate reads. On average, 53% of reads passed alignment QC per individual. Post-QC single-end reads were then extended by 175 bp equilaterally to produce MeDIP fragments of size 225 bp. We quantified coverage of MeDIP fragments per base pair across the genome, and binned coverage per base pair into overlapping 1-kb bins (overlap of 500 bp). For each bin, we calculated the normalized methylation score (RMQ), and in the pain-DMR analyses we only considered bins with RMQ variance >0, where at least 10% of the sample had RMQ scores >0. There were 5,312,714 autosomal 1-kb bins used in the follow-up pain-DMR analyses.

### DMR analyses

Pain-DMR analyses in the discovery MZ twin set were performed using linear mixed-effects models in R (lme4 package). We regressed the RMQ scores in each 1-kb bin on HPST as a fixed effect, family as a random effect, and with and without age as fixed-effect covariate. Before performing the pain-DMR regression, we normalized the RMQ scores to N(0,1), but results were also calculated using the raw RMQ scores (Supplementary Methods). In the discovery set, additional genome-wide pain-DMR analyses also took into account MeDIP-seq fragment variability and CpG density, and only considered specific genomic categories such as CpG-islands, CpG-island shores and promoters (Supplementary Methods). In the second sample of 50 unrelated individuals, we fit linear models regressing RMQ on HPST across the genome, with or without covariates such as age and the first three autosomal methylation principal components, as well as including RMQ normalization (to N(0,1)). The final pain-DMR results (Table 1) include normalized (to N(0,1)) RMQ scores regressed on HPST.

MA of the MZ twin and unrelated samples was performed in GWAMA^56^, using a fixed-effect MA on the HPST pain-DMR regression betas. We report MAP-DMRs at permutation-based MA significance threshold of FDR=5%. We investigated evidence for heterogeneity in the MA using Cochran’s *Q*-statistic and the *I*^2^-statistic^57^, and only considered results with no strong evidence for heterogeneity (Cochran’s *Q*, *P*>0.01 and *I*^2^<0.8) in the observed data and in the permutations. For completeness and to account for heterogeneity, we also performed a random-effects MA and found that the nine FDR 10% MAP-DMRs were within the top-ranked 0.002% of results (Supplementary Table S7). We also checked DMRs for potential mappability problems, but the MAP-DMRs did not overlap previously reported unannotated high-copy-number regions, which may result in alignment problems^58^.

To determine EWAS genome-wide significance thresholds in the discovery and replication samples, we performed 20 replicates of genome-wide permutations. In each replicate, we permuted the HPST scores (preserving family structure in the MZ twin sample) and performed the genome-wide EWAS analyses under the null, and used these to calculate FDR 5% thresholds in the MZ twin and unrelated EWAS. We then combined each pair of MZ twin and unrelated replicates by MA, to determine the MA permutation-based FDR 5% threshold. Genome-wide permutation-based FDR 5% thresholds were estimated for the MZ twin, unrelated and MA results. In each case, FDR was determined as the fraction of significant hits in the permuted data compared with the observed data at each *P*-value threshold. At each nominal *P*-value level, we pooled significant hits across the 20 replicates, divided by the number of replicates (20) and then divided by the number of observed hits in the real data at this nominal *P*-value. There was some variability in FDR estimates at the level of individual replicates, for example, random samples of 10 replicates resulted in MA FDR 5% thresholds ranging from *P*=2.5 × 10^−9^ to *P*=1.0 × 10^−12^, whereas the FDR 5% threshold based on 20 replicates was estimated at *P*=2.0 × 10^−11^.

For within-pair DNA methylation and HPST analyses in the discovery MZ twin set, we calculated the difference in RMQ scores at each 1-kb bin between co-twins in the 25 MZ twin pairs. We calculated Pearson’s and Spearman’s correlations of the RMQ and HPST differences within the MZ twin pair. We also fit a linear model, regressing the normalized RMQ difference on the HPST difference with age as covariate, and the results were similar.

To assess whether the MAP-DMRs identified in our study capture differential proportion of whole-blood cell (WBC) subtypes, we compared DNA methylation with WBC subtype proportions for neutrophils, eosinophils, monocytes and lymphocytes. Blood-count DMR analyses were performed at the nine FDR 5% MAP-DMRs in 48 individuals from the discovery set. We fit a linear mixed-effect model, regressing the normalized RMQ scores on WBC subtype proportion as a fixed effect and family as a random effect. Results are presented at a DMR Bonferroni-corrected *P*-value=0.05 (nominal *P*=0.0056).

### Illumina 450k DNA methylation

Illumina 450k data were obtained for 46 discovery sample individuals. We removed two outliers and performed principal-component analysis, comparing the first two principal components with potential confounders, which identified methylation chip, position of the sample on the chip and post-bisulphite conversion DNA concentration as potential confounders. Therefore, in the pain DMR 450k EWAS analyses, we regressed DNA methylation on HPST levels and included chip, order of sample on the chip, bisulphate-converted DNA concentration and age as fixed effect covariates, and family as random effect. We excluded all probes that mapped to multiple locations within 2 bp mismatches, resulting in 468,113 probes, and in the 450k EWAS analyses we also excluded probes with missing data, which resulted in a 440,307 autosomal probes. We permuted the data ten times to assess significance of the genome-wide 450k EWAS results.

We used two approaches to compare MeDIP-seq and Illumina 450k DNA methylation levels. First, we considered CpG sites that overlapped the 1-kb MeDIP-seq bins (457,045 probes). Second, to validate MeDIP-seq FDR 5% DMR effects in the Illumina 450K results, we considered MeDIP-seq DMRs that were within 100 kb of a gene TSS. We compared the direction of methylation pain association of the MeDIP-seq pain DMR and the direction of methylation pain association of all the 450k CpG sites that were assigned to the same gene. For validation, both MeDIP-seq and 450k DMRs had to be in the same direction and the 450k nominal P surpass weak evidence for association. The DNA methylation data are available at http://www.twinsuk.ac.uk/data-access/medipseq-data/ and under GEO accession number GSE53130.

### Bisulphite pyrosequencing

Bisulphite pyrosequencing DNA methylation in the *TRPA1* DMR genomic region was performed by a commercial laboratory service (EpigenDx). Briefly, 200–500 ng of genomic DNA was used for bisulphite modification using the Zymo Research EZ DNA Methylation Kit (Zymo Research), according to manufacturer’s instructions. Converted genomic DNA was PCR amplified using two sets of *TRPA1* DMR unbiased primers, followed by pyrosequencing using PSQ HS96 (Biotage). Bisulphite pyrosequencing was performed as previously described^59^. The Pyro Q-CpG methylation software (Qiagen) was used to determine the percentage of DNA methylation at each CpG site. Supplementary Table S8 provides the genomic location of the CpG sites measured in the *TRPA1* CpG-island shore DMR. PCR reaction details and annealing temperatures for all bisulfite pyrosequencing reactions are also provided in Supplementary Table S8.

### Genotypes

Genotypes were available for 48 individuals in the discovery sample and for 47 individuals in the follow-up sample. Genotypes were obtained on a combination of Illumina platforms (HumanHap300, HumanHap610Q, 1M-Duo and 1.2MDuo 1M custom arrays) and stringent QC checks were applied to these data as previously described^35^. HapMap genotypes were imputed using Impute (v2 (ref. 60)) with two reference panels, P0 (HapMap2, rel 22, combined CEU, YRI and ASN panels) and P1 (610K+, including the combined HumanHap610K and 1M array). Altogether, there were 2,028,354 (discovery) and 2,054,344 (follow-up) directly genotyped and imputed autosomal single-nucleotide polymorphisms (SNPs), which were further filtered for subsequent analyses.

### Methylation QTLs

DNA meQTL analyses were performed in 47 individuals from the follow-up set. We selected 1,889,799 common HapMap SNPs, either directly genotyped or imputed (Impute info ≥0.8), with minor allele frequency ≥0.05. At each MAP-DMR, we tested for association between DNA methylation levels using RMQ scores in 1-kb bins, and genotypes at all SNPs within 100 kb of the bin (50 kb on either side). We normalized DNA methylation levels (to (N(0,1)) and regressed DNA methylation on genotype using an additive model. Potential MAP-DMRs with meQTLs in *cis* were considered if there was at least one SNP associated with DNA methylation levels at nominal significance, and we report nominal *P*-values of association.

### Allele-specific methylation

ASM was estimated in 48 MZ discovery twins. We obtained heterozygous genetic variants using all available data from directly genotyped and imputed common variants, and exome-sequencing data available for 22 unrelated individuals (44 MZ twins)^61^. In the HapMap-imputed genotypes, we considered SNPs for ASM analysis if Impute info ≥0.8 and if the probability of a heterozygous genotype ≥0.5 in at least 50% of the sample. In the exome-sequencing data, we used direct genotype calls^61^ using a depth threshold of eight and selected variants that were heterozygous in at least 50% of the sample. There were 3,099 heterozygous genetic variants in the exome-sequencing data and 278,304 heterozygous SNPs in the HapMap data, giving an overall final set of 279,885 unique candidate heterozygous genetic variants. For each individual at each of the 279,885 genetic variants, we scored the number of MeDIP-seq reads spanning the reference and non-reference alleles using maq pileup with depth coverage ≥8 reads per variant. We used the frequency of the reference allele in the MeDIP-seq data as a measure of ASM, and scored a site within an individual as ASM only if the reference allele frequency was <0.25 or >0.75. Altogether, potential ASM calls were obtained at 17,261 variants. We excluded all ASM variants that fell in genomic regions reported to be problematic in alignment (Supplementary Methods).

### Blood–brain DNA methylation comparison

MeDIP-seq patterns from blood and multiple brain tissues were obtained from two female donors^37^. The brain regions included the inferior frontal gyrus, middle frontal gyrus, left frontal gyrus, entorhinal cortex, superior temporal gyrus of the temporal cortex, visual cortex and the cerebellum. To assess tissue specificity of methylation effects at the 100 top-ranked MAP-DMRs, we first estimated the correlation between brain and blood methylation levels, by using the mean methylation levels across both individuals in all brain regions and in blood. We then considered regions to have tissue-shared methylation if the methylation difference between blood and one brain region was <10% of the overall blood methylation level, and under more stringent criteria, if the mean difference between blood and all brain region was <10% of the overall blood methylation level (see Supplementary Methods).

### Gene expression

Gene expression data were obtained from skin samples in 341 twins from the MuTHER study^35^, which included MZ and dizygotic twin pairs and unrelated individuals. Gene expression levels were measured using the Illumina expression array HumanHT-12 version 3 as previously described^35^. Each sample had three technical replicates and log_2_-transformed expression signals were quantile normalized first across three replicates of each individual, and second by quantile normalization across all individuals. We used the transformed normalized residuals of the log-transformed gene expression array signal in downstream analysis.

## Author contributions

T.D.S., S.J., J.T.B. and W.J. designed the study. L.M.B., G.F., J.S., H.W. and N.L. performed the experiments. K.W., J.H., S.S., M.D., L.C.S., J.M., F.M.K.W., P.D., S.B. and S.M. contributed reagents, materials and analysis tools. J.T.B. analysed the data with contributions from B.Z., A.K.L. and C.L.H. J.T.B. and T.D.S. wrote the paper. All authors read and approved the manuscript before submission. T.D.S., S.J. and W.J. contributed equally to the study.

## Additional information

**Accession codes:** The sequence and microarray data have been deposited in the NCBI Gene Expression Omnibus under GEO accession number GSE53130. All data sets are also available at http://www.twinsuk.ac.uk/data-access/medipseq-data/.

**How to cite this article:** Bell, J. T. *et al.* Differential methylation of the *TRPA1* promoter in pain sensitivity. *Nat. Commun.* 5:2978 doi: 10.1038/ncomms3978 (2014).

## Supplementary Material

Supplementary Information Supplementary Figures S1-S13, Supplementary Tables S1-S8, Supplementary Methods and Supplementary References

## Figures and Tables

**Table 1:** Meta-analysis DMRs for pain sensitivity at FDR 5%.

				**Discovery (*****n***=25 MZ pairs)*				**Follow-up (*****n***=50 individuals)*				Meta-analysis*		
**Chr**	**DMR**	**Nearest gene (kb)**†	**% Repeat**‡	***β***	**s.e. (*****β*****)**	***P*****-value**	**MZ diff (*****r*****)**§	***β***	**s.e. (*****β*****)**	***P*****-value**	***R***^**2**^	***β***	**s.e. (*****β*****)**	***P*****-value**
8	73,151,000–73,152,000	*TRPA1* (0.1)	0.00	−0.23	0.04	2.6 × 10^−6^	−0.68	−0.40	0.07	1.3 × 10^−6^	0.39	−0.28	0.04	1.2 × 10^−13^
18	5,039,500–5,040,000	NA	0.10	−0.20	0.04	2.6 × 10^−6^	−0.77	−0.27	0.09	3.2 × 10^−3^	0.17	−0.21	0.03	4.1 × 10^−13^
11	123,833,500–123,834,500	*OR8B8* (17.3)	0.30	−0.26	0.04	2.3 × 10^−7^	−0.85	−0.12	0.09	2.0 × 10^−1^	0.03	−0.24	0.03	3.4 × 10^−12^
1	76,264,000–76,265,000	*ST6GALNAC3* (48)	0.23	−0.19	0.03	3.5 × 10^−6^	−0.71	−0.28	0.08	1.6 × 10^−3^	0.19	−0.20	0.03	4.4 × 10^−12^
5	32,273,500–32,274,500	*MTMR12* (0)	0.87	0.24	0.04	2.7 × 10^−5^	0.77	0.34	0.08	8.4 × 10^−5^	0.28	0.26	0.04	5.1 × 10^−12^
4	165,977,000–165,978,000	NA	0.78	0.24	0.04	2.1 × 10^−7^	0.79	0.12	0.09	2.1 × 10^−1^	0.03	0.22	0.03	5.6 × 10^−12^
11	12,054,000–12,055,000	*MICAL2* (33.7)	0.43	−0.27	0.04	5.5 × 10^−8^	−0.59	−0.22	0.09	1.4 × 10^−2^	0.12	−0.26	0.04	7.2 × 10^−12^
1	76,264,500–76,265,500	*ST6GALNAC3* (47)	0.31	−0.18	0.03	5.3 × 10^−6^	−0.68	−0.28	0.09	2.0 × 10^−3^	0.18	−0.20	0.03	1.1 × 10^−11^
2	69,530,500–69,531,500	*NFU1* (12.2)	0.89	−0.24	0.04	8.7 × 10^−7^	−0.68	−0.32	0.08	2.3 × 10^−4^	0.25	−0.26	0.04	1.1 × 10^−11^

DMR, differentially methylated region; FDR, false discovery rate; MZ, monozygote; NA, not applicable; LMER, linear mixed-effects regression; TSS, transcription start site; HPST, heat pain suprathreshold.

^*^Coefficients, s.e. and *P*-values are based on LMER (discovery), linear models (follow-up) and fixed-effect meta-analysis of the two samples.

^†^Nearest gene within 100 kb of the DMR (distance (kb) to nearest TSS from DMR boundary).

^‡^Repeat content estimated as the proportion of ‘N’ bases in each 1 kb DMR bin.

^§^Pearson’s correlation coefficient comparing MZ differences in methylation and HPST.
