# Supplementary material for: Differential methylation of the TRPA1 promoter in pain sensitivity
Source: Nat Commun. 2014 Feb 4;5:2978. doi: 10.1038/ncomms3978 (PMC3926001; doi:10.1038/ncomms3978)
Supplement: Supplementary Information — Supplementary Figures S1-S13, Supplementary Tables S1-S8, Supplementary Methods and Supplementary References [file ncomms3978-s1.pdf]

Supplementary Information: Differential methylation of the *TRPA1* promoter in pain sensitivity

Bell JT, Loomis AK, Butcher LM, Gao F, Zhang B, Hyde CL, Sun J, Wu H, Ward K, Harris J, Scollen S, Davies MN, Schalkwyk LC, Mill J, The MuTHER Consortium, Williams FMK, Li N, Deloukas P, Beck S, McMahon SB, Wang J, John S, and Spector TD.

Supplementary Figures

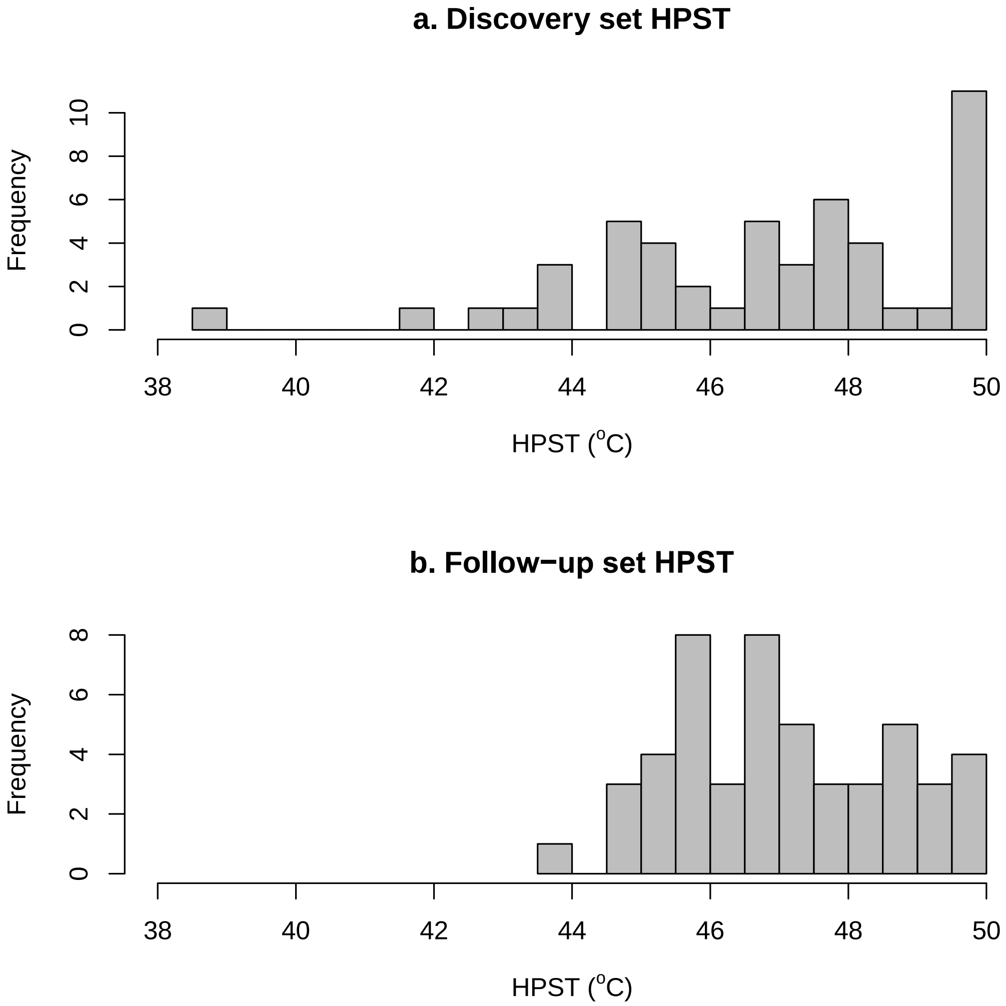

Supplementary Figure S1. Heat pain suprathreshold (HPST) distribution in the (a) discovery monozygotic twins and (b) follow-up unrelated samples.

MeDIP-seq analysis in the Discovery sample

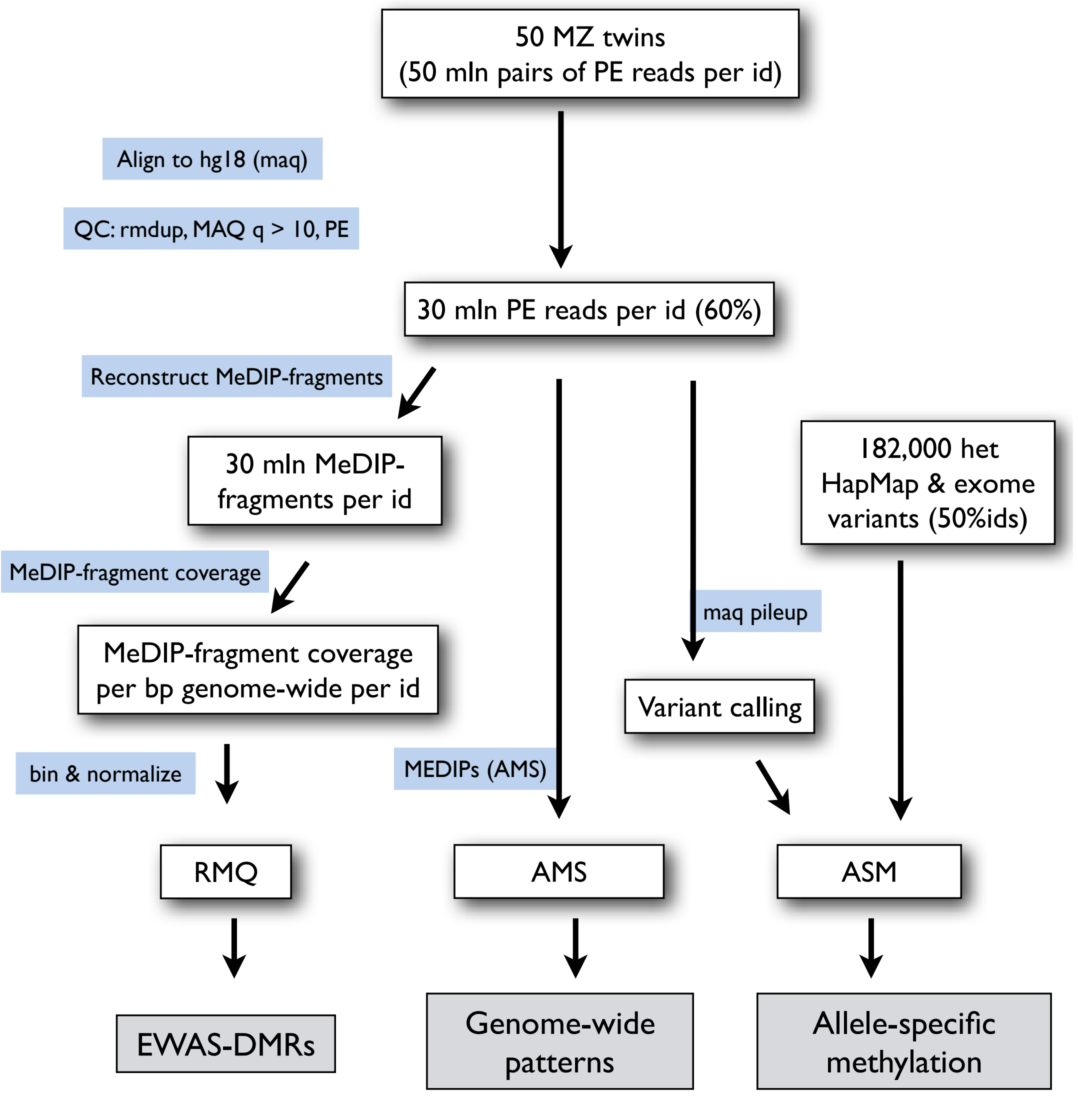

Supplementary Figure S2. Overview of the MeDIP-sequencing quantification analysis in the discovery MZ twin sample.

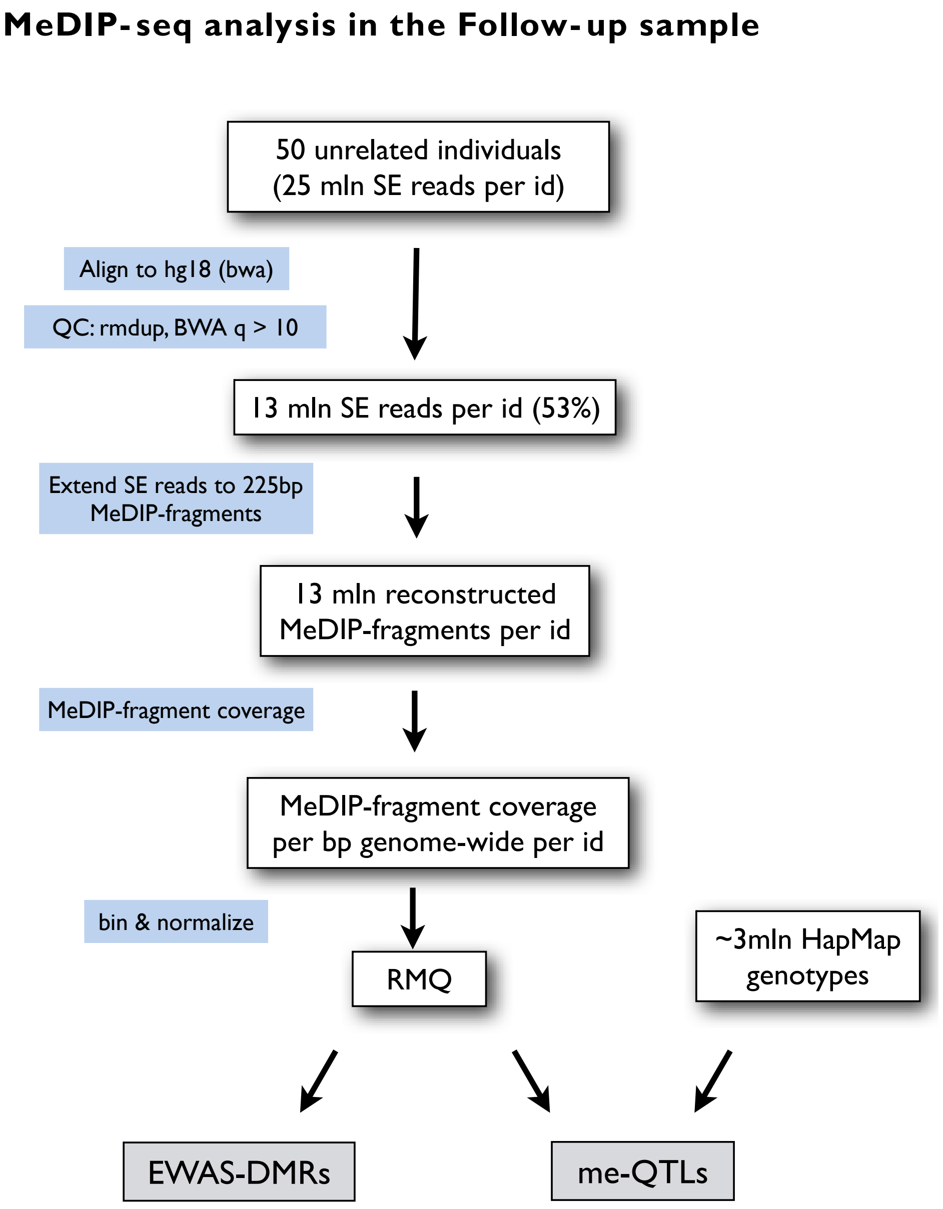

Supplementary Figure S3. Overview of the MeDIP-sequencing quantification analysis in the follow-up unrelated sample.

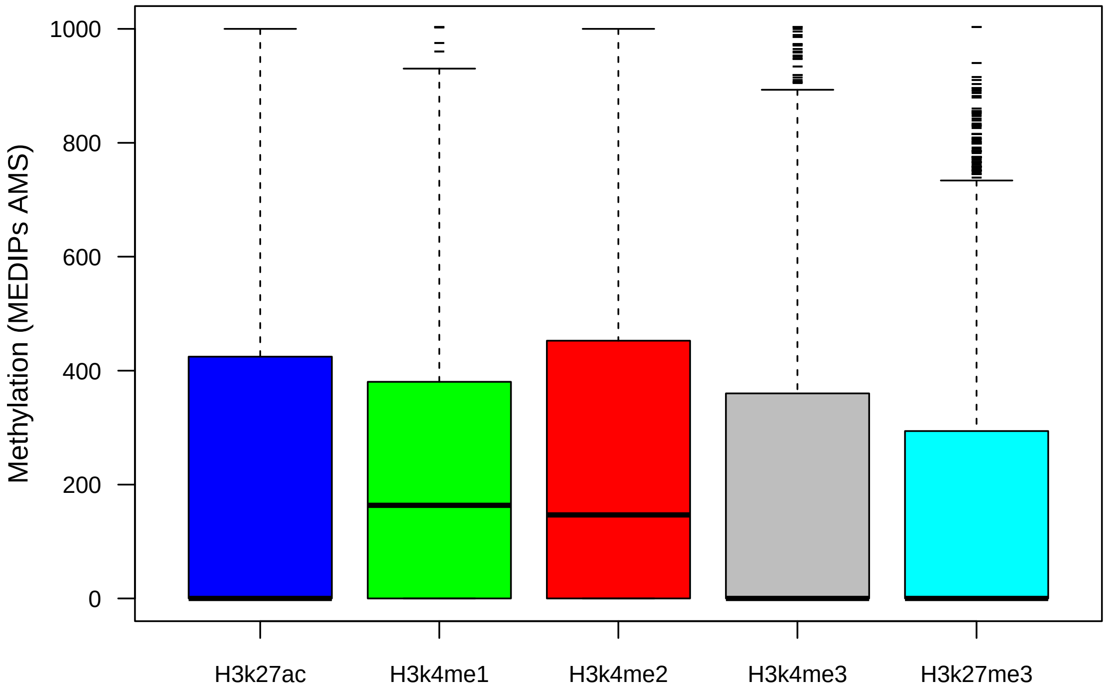

Supplementary Figure S4. DNA methylation levels in genomic regions that overlap histone modification peaks for 5 histone marks in one lymphoblastod cell line sample from the Encode project. Boxes show the 25% and 75% quantiles and whiskers extend to 1.5 times the IQR.

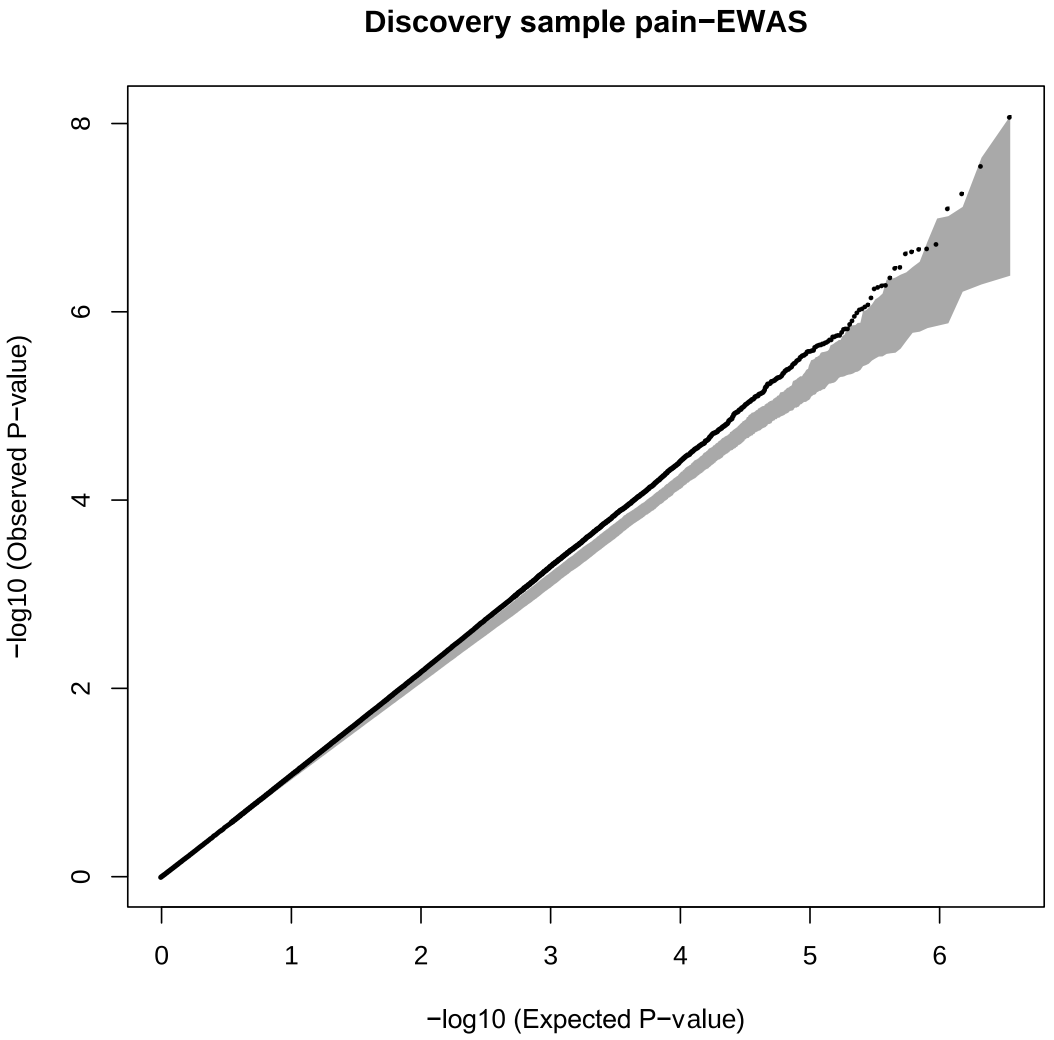

Supplementary Figure S5. MZ twin EWAS results. Quantile-quantile (QQ) plot describing the enrichment of association signal in the pain-sensitivity EWAS, compared to the permuted data (permutation-based 95% confidence band shaded).

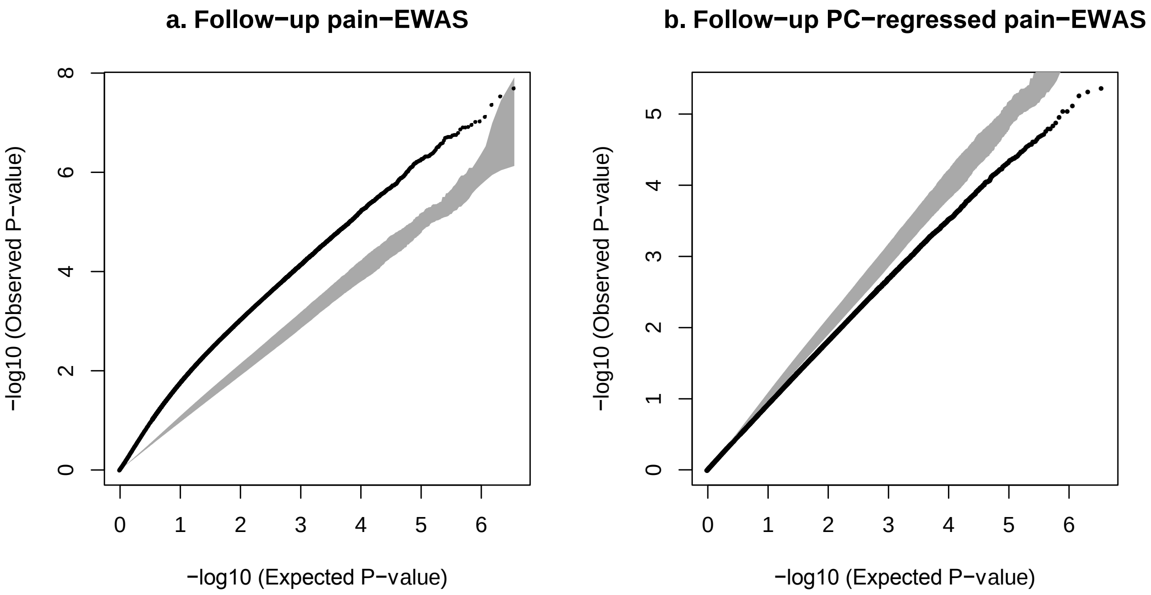

Supplementary Figure S6. Follow-up unrelated-sample EWAS results. (a) Association results from a linear model regressing normalized methylation levels on pain scores, compared to the permuted data (permutation-based 95% confidence band shaded). (b) Association results from a linear model regressing normalized methylation levels on pain scores, including the first three autosomal principal components as covariates. The first three PCs together explained 25.7% of the variance in methylation.

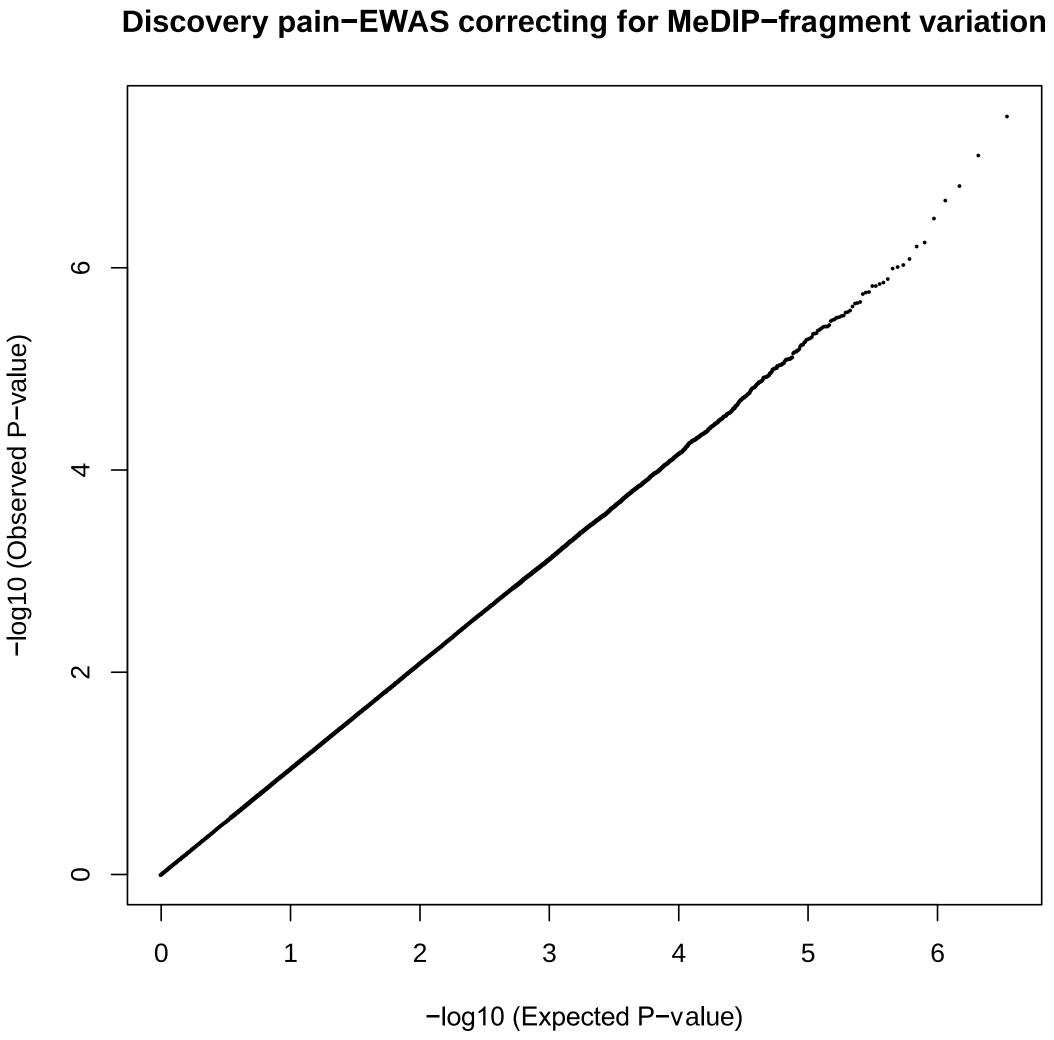

Supplementary Figure S7. Discovery MZ twin EWAS results, controlling for MeDIP-fragment size variability.

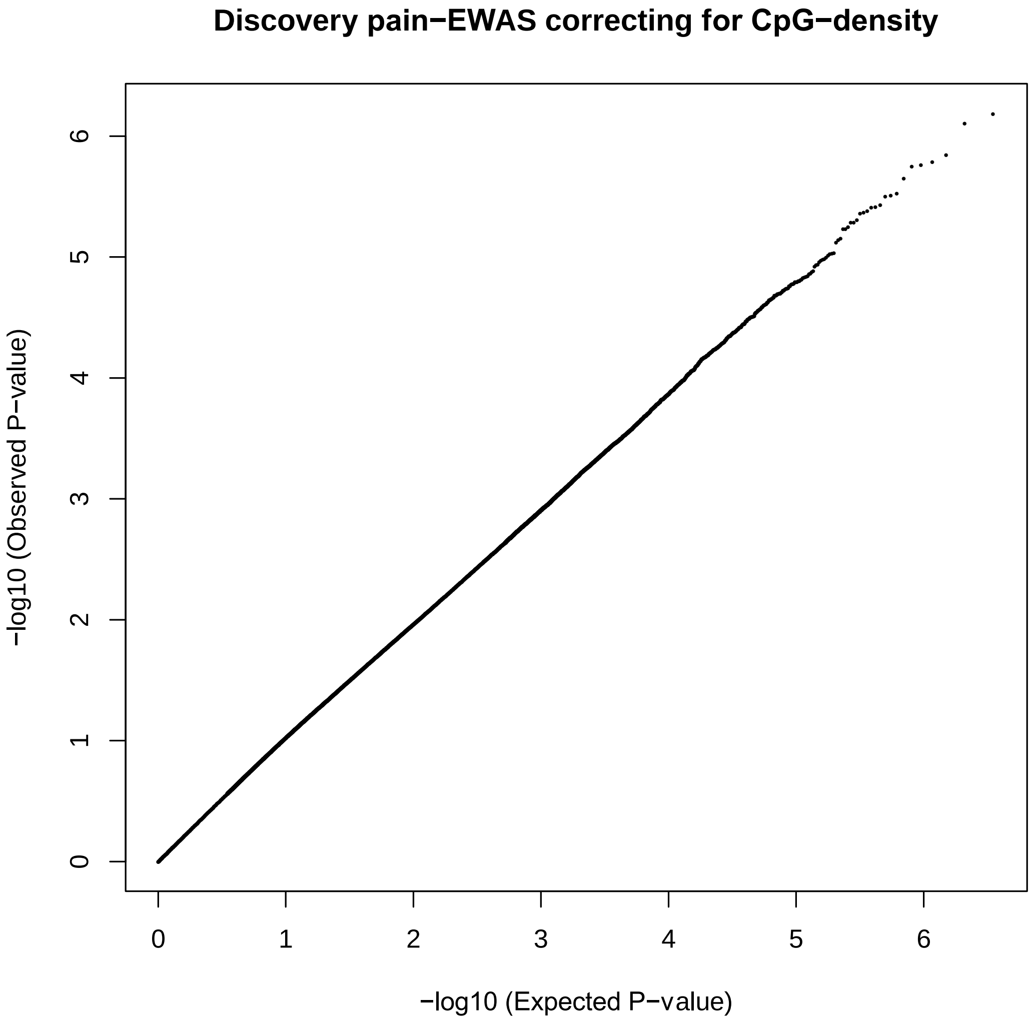

Supplementary Figure S8. Discovery MZ twin EWAS results, controlling for CpG-density using MEDIPs AMS methylation quantification scores.

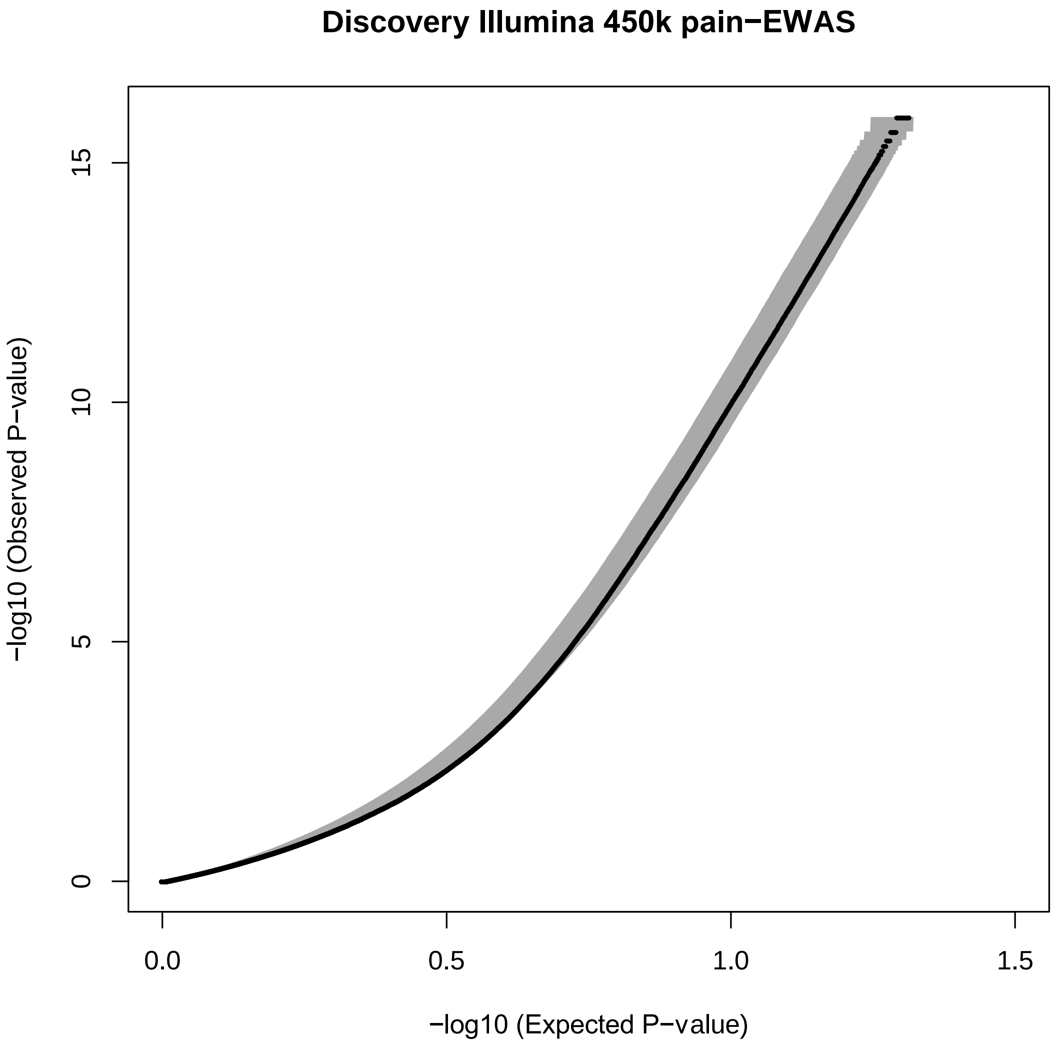

Supplementary Figure S9. Discovery MZ twin Illumina 450k EWAS results, compared to the permuted data (permutation-based 90% confidence band shaded).

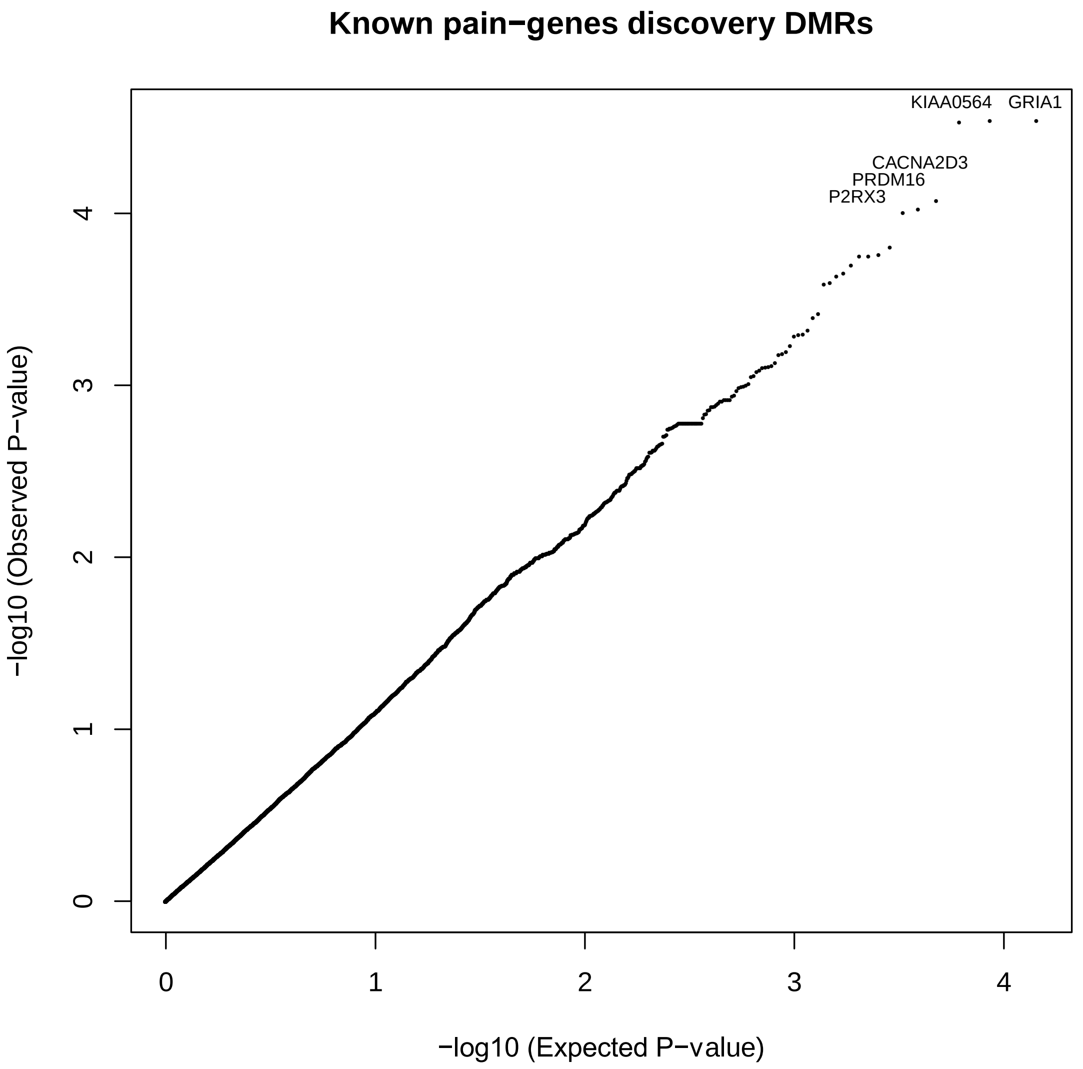

Supplementary Figure S10. DMR effects in 73 known candidate genes for pain in the discovery set of 50 MZ twins.

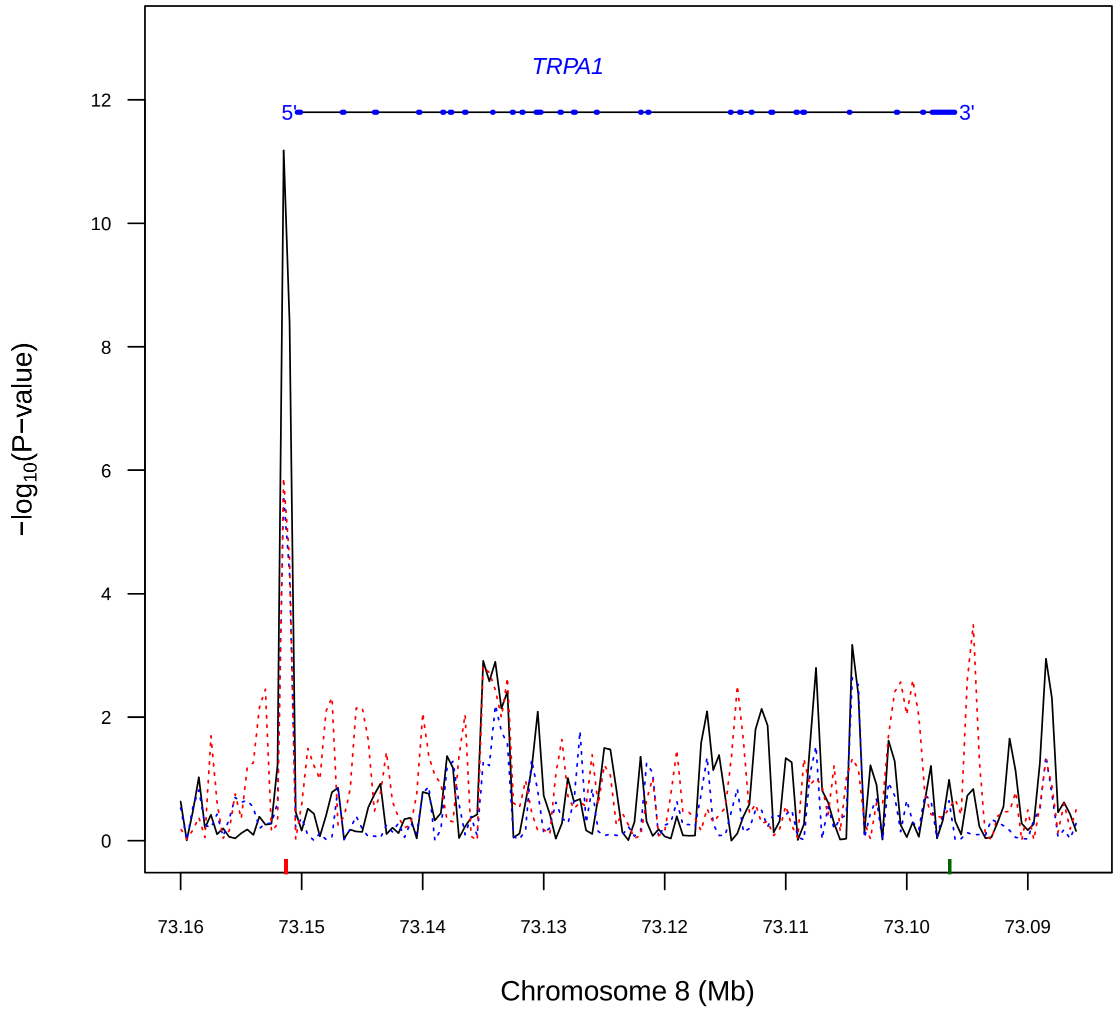

Supplementary Figure S11. Differential methylation in TRPA1 in pain sensitivity. Association results in the TRPA1 gene region (negative strand) in the meta-analysis (black solid line) and in the discovery (blue dotted) and follow-up (red dotted) samples. Bisulfite pyrosequencing was performed in the pain-DMR promoter region (red ticks), and data for one gene expression probe was available located near the TES (green tick).

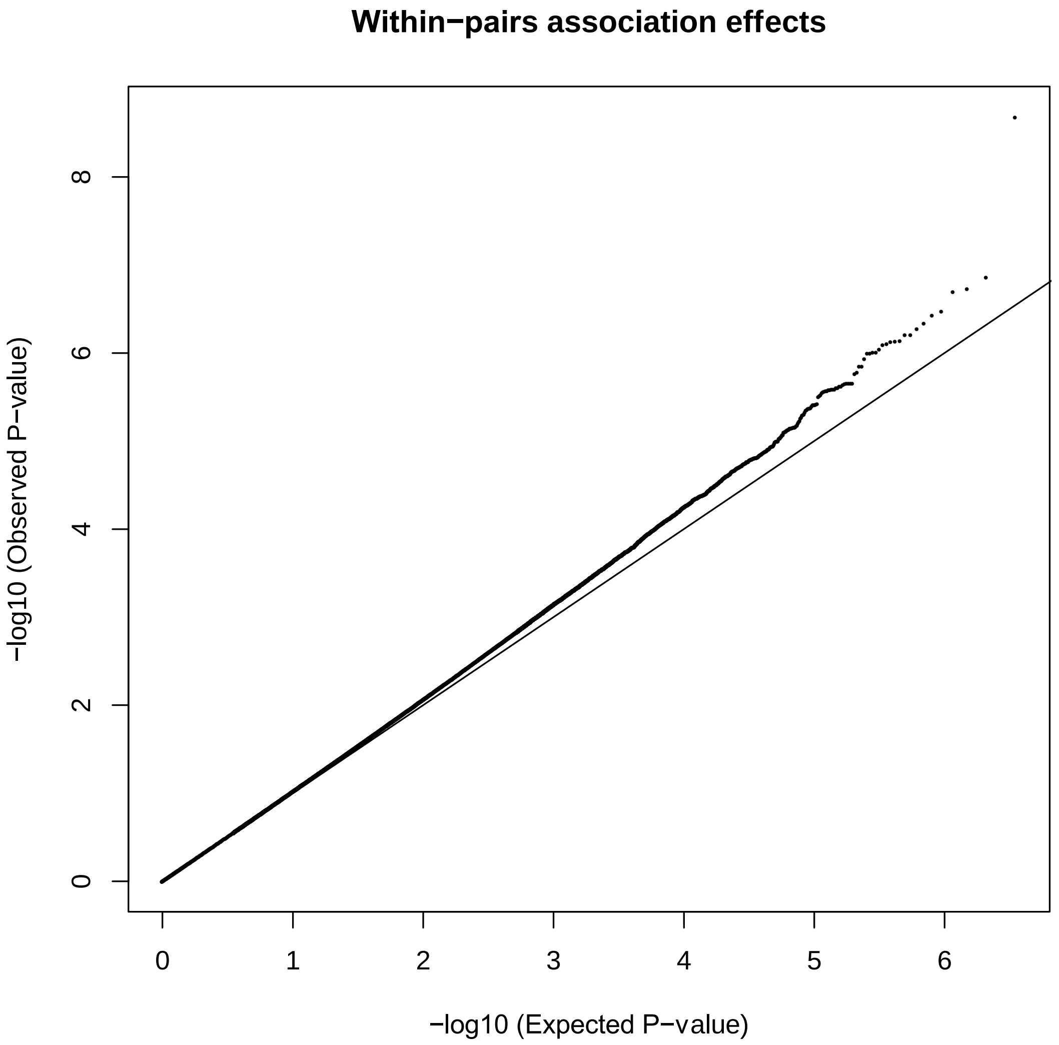

Supplementary Figure S12. Within-pair DMRs in the discovery MZ twin set. P values are the rank correlation P-values comparing the HPST differences to methylation differences within MZ twin pairs.

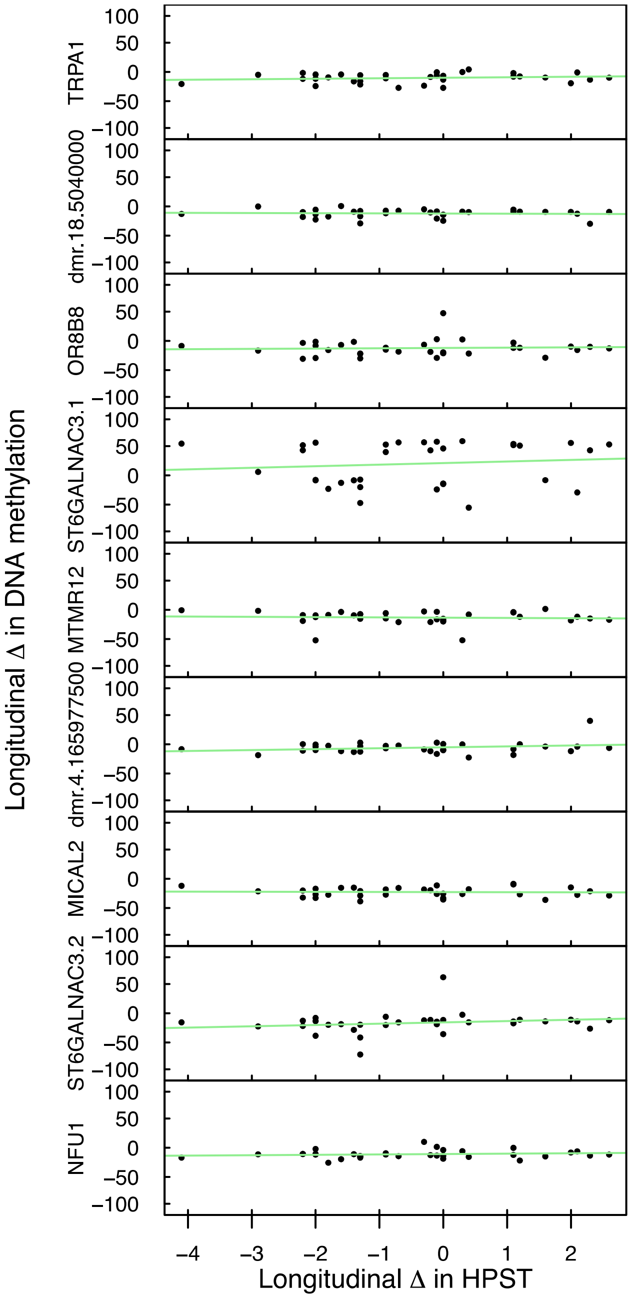

Supplementary Figure S13. Longitudinal stability of the 9 FDR 5% MAP-DMRs. Longitudinal methylation and HPST effects at 9 MAP-DMRs in 33 individuals are c onsistent with stable methylation over time.

Supplementary Tables

Supplementary Table S1. Top ten discovery EWAS DMRs, of which only the top hit surpassed FDR 5% for the LMER analyses.

Rank	Chr	Mid-point	Beta	SE	P-value	Nearest-gene	Distance-to-TSS
1	3	173116500	-0.28	0.040	8.41e-09	NA	NA
2	10	80783000	-0.27	0.038	2.80e-08	PPIF	0
3	11	12054500	-0.27	0.041	5.50e-08	MICAL2	33713
4	16	29745500	0.26	0.042	7.90e-08	MVP	0
5	7	143856000	-0.24	0.040	1.89e-07	TPK1	0
6	2	8523500	-0.24	0.040	2.10e-07	NA	0
7	4	165977500	0.24	0.035	2.13e-07	NA	NA
8	11	123834000	-0.26	0.037	2.28e-07	OR8B8	17309
9	12	115083500	-0.23	0.039	2.37e-07	MED13L	0
10	3	158246000	-0.25	0.043	2.76e-07	EBF1	0

Supplementary Table S2. Follow-up EWAS DMRs

Rank	Chr	Mid-point	Beta	SE	P-value	Nearest-gene	Distance-to-TSS
1	10	61642500	-0.45	0.07	1.96E-08	ANK3	0
2	5	168670500	-0.45	0.07	2.87E-08	SLIT3	9289
3	8	81823500	-0.45	0.07	4.22E-08	ZNF704	0
4	15	56013500	-0.44	0.07	7.401E-08	ALDH1A2	79527
5	8	61343000	-0.44	0.07	9.12E-08	CA8	0
6	20	48008500	0.44	0.07	9.43E-08	RNF114	21680
7	17	41076000	0.44	0.07	1.06E-07	C17orf69	0
8	6	117981500	-0.43	0.07	1.19E-07	DCBLD1	0
9	13	35550000	-0.43	0.07	1.22E-07	DCLK1	0
10	2	201320500	-0.43	0.07	1.22E-07	AOX2P	22639
11	2	100171500	-0.43	0.07	1.33E-07	AFF3	45531
12	8	36704000	-0.43	0.07	1.57E-07	KCNU1	56499
13	10	86473500	-0.43	0.07	1.71E-07	NA	100000
14	9	123722000	-0.43	0.07	1.73E-07	TTLL11	0
15	14	78829000	-0.43	0.07	1.77E-07	NRXN3	0
16	15	37283000	-0.43	0.07	1.87E-07	C15orf54	46676
17	12	120646500	0.43	0.07	1.88E-07	TMEM120B	0
18	4	23570500	-0.43	0.07	1.90E-07	PPARGC1A	69202
19	13	53292500	-0.43	0.07	1.92E-07	NA	100000
20	20	2588500	0.43	0.07	1.95E-07	IDH3B	0

Supplementary Table S3. Follow-up PC-regressed top EWAS results.

Rank	Chr	Mid-point	Beta	P-value	Nearest-gene	Distance-to-TSS
1	5	26294500	0.39	4.25E-06	NA	100000
2	2	188537500	-0.39	4.76E-06	NA	100000
3	8	61343000	-0.39	5.40E-06	CA8	0
4	2	34733500	-0.38	7.519E-06	NA	100000
5	16	8205000	-0.38	8.90E-06	NA	100000
6	17	41076000	0.38	9.01E-06	C17orf69	0
7	21	32647000	0.38	1.08E-05	URB1	0
8	22	32513000	-0.37	1.31E-05	LARGE	0
9	10	73791500	0.37	1.44E-05	DNAJB12	6087
10	4	55929500	0.37	1.57E-05	SRD5A3	0
11	14	80609000	-0.37	1.58E-05	TSHR	0
12	15	56013500	-0.37	1.71E-05	ALDH1A2	79527
13	10	61642500	-0.37	1.89E-05	ANK3	0
14	13	53292500	-0.37	1.95E-05	NA	100000
15	14	41627000	0.37	2.02E-05	NA	100000
16	11	132306500	-0.37	2.08E-05	OPCML	0
17	20	2588500	0.37	2.23E-05	IDH3B	0
18	3	56247000	-0.36	2.392E-05	ERC2	0
19	20	24399000	-0.36	2.41E-05	C20orf39	0
20	2	51625500	0.36	2.42E-05	NA	100000
21	4	184967000	0.36	2.59E-05	STOX2	96002
22	11	29090500	-0.36	2.62E-05	NA	100000
23	5	219000	0.36	2.65E-05	PLEKHG4B	0
24	3	33744500	0.36	2.76E-05	CLASP2	9148
25	5	113648000	0.36	2.78E-05	KCNN2	77414
26	13	35550000	-0.36	2.89E-05	DCLK1	0
27	6	117981500	-0.36	3.00E-05	DCBLD1	0
28	10	58047500	-0.36	3.13E-05	NA	100000
29	8	81823500	-0.36	3.17E-05	ZNF704	0
30	8	9503000	-0.36	3.30E-05	TNKS	0
31	9	4333000	-0.36	3.33E-05	GLIS3	42465
32	10	86473500	-0.36	3.338E-05	NA	100000
33	2	45337000	-0.36	3.48E-05	NA	100000
34	4	182018000	-0.36	3.54E-05	NA	100000
35	12	1849500	0.36	3.64E-05	CACNA2D4	0

Supplementary Table S4. Meta-analysis top-ranked results with PC-regressed follow-up estimates.

Rank	Chr	DMR-bp-midpoint	beta	P-value	Nearest-gene	Distance-to-TSS
1	18	5040000	-0.20	0.0000000000345	NA	100000
2	1	76264500	-0.19	0.0000000000856	ST6GALNAC3	47976
3	1	76265000	-0.18	0.000000000162	ST6GALNAC3	47476
4	2	8523500	-0.23	0.000000000176	NA	100000
5	8	73151500	-0.24	0.000000000305	TRPA1	627
6	5	32274000	0.24	0.000000000627	MTMR12	0
7	4	30456000	0.22	0.00000000106	PCDH7	0
8	11	12054500	-0.23	0.00000000106	MICAL2	33713
9	14	50273500	-0.21	0.00000000127	NIN	0
10	2	69531000	-0.23	0.00000000241	NFU1	12243
11	1	210710500	-0.23	0.00000000792	NENF	37149
12	12	128329000	-0.19	0.000000011	TMEM132D	0
13	6	161082500	-0.20	0.0000000115	PLG	0
14	1	4586000	-0.17	0.0000000131	AJAP1	28464
15	2	223607000	-0.21	0.0000000168	KCNE4	17605
16	7	100711500	-0.17	0.0000000173	FIS1	35909
17	2	210490500	-0.20	0.0000000226	C2orf21	0
18	18	12447000	-0.20	0.0000000258	SPIRE1	0
19	6	163968500	-0.20	0.0000000293	NA	100000
20	14	86896500	-0.22	0.0000000306	NA	100000
21	5	21648000	-0.20	0.0000000307	NA	100000
22	12	32988500	-0.21	0.0000000318	PKP2	46953
23	15	60248500	-0.20	0.0000000323	FAM148B	3226
24	13	24625500	-0.20	0.0000000336	FAM123A	17857
25	11	108268000	-0.23	0.0000000339	DDX10	0
26	2	99159000	0.20	0.0000000363	MITD1	0
27	5	56391500	0.20	0.0000000373	NA	100000
28	14	22253500	-0.22	0.0000000387	OXA1L	51570
29	13	52603500	-0.22	0.0000000398	NA	100000
30	5	112378000	-0.18	0.00000004	DCP2	0

Supplementary Table S5. meQTL results (linear model meQTL $P < 0.05$) at 9 MAP-DMRs.

Rank	Chr	DMR-bp-midpoint	Nearest-gene	SNP	SNPbp	P-value
1	8	73151500	TRPA1	rs2383849	73166858	0.032520915
2	18	5040000	NA	rs8084399	5087618	0.034106905
3	11	123834000	OR8B8	rs12282735	123818112	5.81E-06
4	1	76264500	ST6GALNAC3	rs10493579	76275783	0.000140576
6	4	165977500	NA	rs11722903	165959777	0.010227358
8	1	76265000	ST6GALNAC3	rs10493579	76275783	3.01E-05

Supplementary Table S6. Longitudinal results at 18 MAP-DMRs in Figure 3B. HPST-MethStable refers to the mean longitudinal HPST change per individual, in the group of individuals where DNA methylation changed by less than 30% over time. HPST-MethVar refers to the mean longitudinal HPST change per individual, in the group of individuals where DNA methylation changed by greater than 30% over time. The P-value is the T-test p-value comparing the mean HPST in the two groups (MethStable and MethVar).

Name	Chr	DMR-bp-midpoint	HPST-MethStable	HPST-MethVar	P
MICAL2.DMR*	11	12054500	-0.40	-0.65	0.72
PKP2.dmr	12	32988500	-0.27	-0.83	0.37
PCDH7.dmr	4	30456000	0.35	-1.11	0.008
MVP.dmr	16	29745500	-0.36	-0.69	0.631
DTNA.dmr	18	30663000	-0.26	-0.55	0.65
dmr.11.79815500	11	79815500	0.05	-0.69	0.20
C1orf168.dmr	1	57015000	-0.21	-0.72	0.36
dmr.13.52604000	13	52604000	-0.40	-0.59	0.75
FAM33A.dmr	17	54551500	-0.30	-0.99	0.18
dmr.7.84180000	7	84180000	-0.39	-0.55	0.79
GLYATL1.dmr	11	58469000	-0.13	-0.74	0.28
dmr.1.179605000	1	179605000	-0.26	-1.28	0.03
dmr.15.51454500	15	51454500	-0.21	-0.81	0.30
FARP1.dmr	13	97860500	-0.31	-0.85	0.52
TMTC2.dmr	12	81842500	-0.19	-0.88	0.27
KCNE4.dmr	2	223606500	-0.39	-0.6	0.70
LOC100216001.dmr	10	4703500	-0.41	-0.51	0.87
FSHB.dmr	11	30252000	-0.26	-0.64	0.50

Supplementary Table S7. Random-effects meta-analysis results for the top 9 MAP-DMRs.

Fixed-Rank	Chr	DMR-bp-midpoint	Fixed-MA P-value	Random-MA P-value	Nearest-gene
1	8	73151500	1.2E-13	3.6E-04	TRPA1
2	18	5040000	4.1E-13	4.1E-13	NA
3	11	123834000	3.4E-12	7.5E-04	OR8B8
4	1	76264500	4.4E-12	2.9E-08	ST6GALNAC3
5	5	32274000	5.1E-12	5.9E-09	MTMR12
6	4	165977500	5.6E-12	2.06E-04	NA
7	11	12054500	7.2E-12	7.15E-12	MICAL2
8	1	76265000	1.1E-11	1.56E-07	ST6GALNAC3
9	2	69531000	1.1E-11	1.10E-11	NFU1

Supplementary Table S8. Bisulfite pyrosequencing reaction details.

Assay	PCR size	No CpGs
ADS2307FS1	183bp	2
ADS2308FS1	225bp	2
ADS2308FS2	225bp	3

Sequences analysed:

ADS2307FS1: YGTTGTTGAGTGTATTTATGTTAGT/GTTTTGGATTTAYGGGGGTTTTGAG TTAAGTGTTGG

ADS2308FS1: TTTTTTGYGTTTTAGTTTTGTAAATTTGAATTTAYGGTAATAGTTTTTTTG

ADS2308FS2: TTTTTTTGTTTGTATTAGATAGTTTTTTTGTTTGTAYGTAAAAG-TAGAATTAGTTTTTTYGAT TGAGATATTTYGATTGATATTTAATGAAGAG

Supplementary Methods

Sample selection and phenotyping

The individuals included in the study were unselected twin volunteers from the TwinsUK cohort, who were recruited through national media campaigns and from other twin registers. Twins from this cohort have been shown to be comparable to the age-matched general population singletons for a broad variety of medical and behavioural traits[62]. Subjects were invited to attend St Thomas Hospital where they completed questionnaires gathering demographic information, clinical history and current medications. Exclusions for this study included volunteers who had consumed analgesic medication within 12 hours of the study visit, and those with likely impaired upper limb neurology, for example, known neuropathy, previous stroke or chemotherapy. Subjects with common painful conditions such as osteoarthritis were not excluded.

Twins underwent quantitative sensory testing (QST) individually, with the co-twin absent from the room while the test was performed, as previously described[61]. Briefly, each subject was seated at a table onto which the test arm was placed. A 25mm2 x 50mm2 probe connected to a Modular Sensory Analyzer Thermal Stimulator (Somedic, Sweden) was secured with a fabric-covered band on the volar surface of the forearm. Subjects received standardized instructions before assessment. The heat pain supra threshold (HPST) represents the temperature at which the sensation evoked by a thermal stimulus changes from ”painful” to ”unbearable”. HPST was measured by heating the probe (at 10C/s) from an adaptation temperature of 320C until the subject perceived the stimulus as changing from painful to unbearable and stopped the experiment, at which point the temperature (equivalent to HPST) was automatically logged. If the probe reached 500C the machine automatically returned to adaptation temperature to prevent thermal burn. Blood was taken for DNA extraction after QST had been performed.

In the discovery sample we selected twenty-five monozygotic (MZ) female twin-pairs (age range 46-76, median age 62) from TwinsUK. The 25 discovery MZ twin pairs were selected as the most discordant MZ twin pairs for HPST in the TwinsUK cohort at the time of the study (Supplementary Fig. 1). Discordance was defined as a difference of at least 20C in HPST scores, and one twin in each pair fell in the upper tail of the HPST distribution. The HPST MZ intraclass correlation coefficient was -0.144 in these data, which are selected for discordance.
The follow-up sample consisted of 50 unrelated individuals (age range 42-86, median age 63.6) was ascertained separately 2-3 years after the discovery sample. The follow-up unrelated sample was unselected for HPST score distribution (Supplementary Figure 1).

White blood cell (WBC) sub-type counts were obtained in 48 individuals from discovery sample using fluorescence activated cell sorting of peripheral blood 3. WBC sub-type cell counts were calculated by multiplying the proportion of the WBC count comprised by each cell type by the total WBC cell count (estimated in thousands of cells per ml), for four cell types in our sample: neutrophils, eosinophils, monocytes, and lymphocytes.

MeDIP-seq DNA methylation

MeDIP-seq Data Quality Control

We used Maq (v0.7.1) in the discovery MZ sample and BWA (v0.5.9) in the follow-up unrelated sample to align the sequence reads to the human genome (hg18), excluding clones that are not yet finished or cannot be placed with certainty at a specific place on the chromosome (files that were labelled ”random”). The alignment was performed with the default parameters in Maq and BWA.

We then assessed the quality of the read alignments, fragment location, and depth of coverage. We only considered reads that mapped at stringent alignment mapping thresholds (Maq q > 10 in discovery set; BWA q >10 in follow-up set) and excluded duplicated reads. We also only considered reads that were assigned as properly paired in the discovery set.

Quantifying MeDIP-seq DNA methylation levels

In the discovery set, we used the post-QC paired-end reads to determine the size and location of the corresponding MeDIP fragment, where the average fragment size was 236bp. We first obtained the per base-pair coverage across the genome, where each base that overlapped a mapped MeDIP fragment was given a score of +1. This constitutes the read-depth distribution. We obtained the read-depth distribution per individual including both lanes for that individual. We then binned coverage sum in overlapping windows of 1kb (overlap of 500bp).

Illumina 450k DNA methylation

We used Illumina 450K methylation profiles for 44 individuals from the discovery sample. We initially obtained Illumina 450k unprocessed beta values for 46 individuals from the discovery sample for 485,837 Illumina 450k probes. At each CpG-site for each individual the beta value represents the ratio of methylated bead signals over the sum of the methylated and unmethylated bead signals from the probe on the array. Illumina beta values range between 0 (unmethylated) and 1 (methylated).

We mapped the 50bp Illumina 450K probe sequences to the human genome (hg18) to confirm probe locations and check for sequence mismatches. We excluded all probes that mapped to multiple locations in the genome with up to 2 mismatches. Altogether, we excluded 17,651 probes. Furthermore, we only considered autosomal probes with no missing data in our sample, which resulted in a final number of 424,368 autosomal probes for further analysis.

We examined the individual-level Illumina 450k profiles for the presence of outliers and to identify potential confounders. The distributions of the unprocessed beta profiles across the genome highlighted two individuals who were outliers, and these were subsequently removed from the analyses.We then performed principal component analysis (PCA) of the Illumina 450k data and compared the first PC loadings (the first PC explained 22% of the variance) to potential confounders, and found associations with methylation chip, the

concentration of the bisulfite converted DNA, and a weak association with position of the sample on the chip. Therefore, we included these three terms as covariates in the phenotype analyses.

Altogether, Illumina 450K CpG-sites overlapped 422,158 overlapping 1kb regions used in the MeDIP-seq quantifications.

Bisulfite pyrosequencing

Bisulfite pyrosequencing of 250-300bp regions was performed by EpigenDx laboratory service. The methylation status of each CpG site was analyzed individually as an artificial TC single nucleotide polymorphism using Q CpG software (Qiagen Pyrosequencing, Valencia, CA). The methylation level at each CpG site for each sample was calculated as the percentage of the methylated alleles over the sum of the methylated and unmethylated alleles. The mean methylation level was calculated using methylation levels of all measured CpG sites within the PCR region of a gene. The methylation level at each CpG site ranges from 0 (unmethylated) to 1 (fully methylated). EpigenDx performed the assay validation, bisulfite conversion, and pyrosequencing. We included high, medium, and low methylated DNA as controls in each run.

Controls included low methylated control DNA and in vitro methylated DNA were mixed at different ratios (0, 20, 40, up to 100%) followed by bisulfite modification, PCR, and pyrosequencing analysis. The percent methylation obtained from the mixing study was highly correlated with an R2 value of 0.99. The assay was also validated by sensitivity and reproducibility testing. Different amounts of genomic DNA were used for the analysis in triplicates. Reproducible results were obtained by using as low as 5 ng of genomic DNA in the bisulfite modification reaction.

Please refer to Supplementary Table S8 for primer and sequence details. The PCR Cycling conditions were as follows:
ADS2307 950C 15 min; 45 x (950C 15 s; 560C 30 s; 720C 30 s); 720C 5 min; 40C inf
ADS2308 950C 15 min; 45 x (950C 15 s; 600C 30 s; 720C 30 s); 720C 5 min; 40C inf

Pain-DMR analysis

Discovery MZ-twin MeDIP-seq pain-DMRs

In the discovery sample we used a linear mixed effects model where we fit the temperature-sensitivity of each individual as a function of methylation at a given 1kb locus. At each locus the methylation data were quantile normalised (to N(0,1)) prior to the analyses. We also ran the analyses by including age as a fixed effect covariate, but the top-ranked results were very similar to the non-corrected results (ranks varied by at most 5 units in either direction). We also ran the analyses by normalising the RMQ scores by the overall number of post-QC reads assigned to each chromosome, and observed that the top-ranked results were also very similar.

To further assess the sensitivity of pain-DMRs to methylation quantification of MeDIP-seq data we also performed analyses controlling for MeDIP-seq fragment size and CpG

density (using MEDIPS AMS) in the discovery EWAS (Supplementary Figures 7-8). The majority of the top-ranked pain-DMRs remained nominally significant in the additional analyses, although the relative rank could change. For example, the *TRPA1* pain-DMR (originally ranked 50th, P = 2.6 x 10-6) was nominally significant when controlling for MeDIP-seq fragment size (ranked 49th, P = 2.6 x 10-6) and CpG-density (ranked 36,921st (in top 0.07%), P = 7.8 x 10-3). Further detail on these additional analyses is provided in the following two paragraphs.

The first additional analysis corrected for MeDIP-fragment size variation across the 50 individuals (Supplementary Figure 7). We compared the distributions of the proportion of the MeDIP-fragments at each given size from 50 to 350bps. There were 4 outliers in the data, which we removed. We then took the minimum proportion of MeDIP-fragments per size category and used this distribution as the distribution to normalize each individual to. For each individual we then dropped fragments at random within size category, until the MeDIP-fragment by size distribution matched the normalisation distribution. We used this subset of MeDIP-fragments in the downstream quantifications and analyses, which were performed as described for the main (RMQ) analyses.

The second additional DMR analysis in the discovery set took into account local CpG-density in the DNA methylation quantifications (Supplementary Figure 8). We used MEDIPs to calculated absolute methylation scores (AMS), which we then used in downstream analyses as described for above. We excluded all bins where MEDIPS was not able to estimate discrete AMS (score was Infinity), or where fewer than 10% of individuals had methylation levels > 0. In the regression model, we also quantile normalised (to N(0,1)) the AMS scores in each 1kb bin. In general, the RMQ analyses were more similar to the analyses correcting for fragment-size, than the CpG-density correction. However, in the CpG-density weighted results the ranks of the top ranked RMQ DMRs showed greater variability, although the majority of the top 100 RMQ DMRs remained nominally significant.

We also compared the within-pair signed differences between pain-sensitivity and degree of methylation at a given locus within each MZ twin pair. The phenotype data here represented the difference in temperature-sensitivity between the pain-insensitive and pain-sensitive twin (therefore, the phenotype differences were always positive). The methylation data represented the corresponding difference in methylation quantification, assessed in tiles of 1000bp, between the pain-insensitive and pain-sensitive twin. To compare the methylation to phenotype differences, we applied a moderated t-test and a non-parametric Mann-Whitney U test to these data.

Follow-up unrelated MeDIP-seq pain-DMRs

In the follow-up unrelated sample, we ran the initial analyses by comparing normalised (to N(0,1) RMQ scores against HPST for each bin in the genome, using linear models. We also performed these analyses including age as covariate, without normalising (to N(0,1)) the RMQ scores and observed that the top ranked results had very similar ranks, effects and P-values in the additional analysis. We observed an inflation of test statistics overall in the follow-up results, and to explore this further we also repeated the analyses by including the first up to 3 principal components in the autosomal methylation data in the

follow-up samples, which together explained 25.7% of the variance.

Discovery MZ-twin BiSeq pain-DMRs

We performed BiSeq validation of the *TRPA1* region. In the pain-DMR analyses with the BiSeq data, we validated the MeDIP-seq signal at two CpG-sites at chr8:73151235bp (Pain-DMR rank correlation = 0.22, and association taking into account twin structure P = 0.03), and at chr8:73151380bp (Pain-DMR rank correlation = 0.67, and association taking into account twin structure P = 0.025).

Allele specific methylation (ASM) analysis

ASM analyses were performed only in the discovery set of MZ twins because MeDIP-seq coverage was greater in these data (about 50mln paired-end reads per individual). Briefly, we obtained a subset of heterozygous genetic variants from genotype and exome sequencing data in 48 MZ twins, and for each individual at each genetic variant we scored the number of MeDIP-seq reads spanning the reference (R) and non-reference alleles. We used the frequency of the reference allele in the MeDIP-seq data as a measure of ASM at each heterozygous genetic variant.

We merged exome sequencing data and directly genotyped and imputed (to hapmap2) genotype data to obtain a set of heterozygous SNPs for ASM analysis. For exome sequencing data we used genotype calls according to the analysis described in reference 61. For hapmap2 imputed genotypes, we required that the Impute2 info threshold of the SNP surpassed 0.8 and that the probability of a heterozygous genotype surpassed 0.5.
We then selected all SNPs where at least 50% of individuals were heterozygous (13 of 24 MZ pairs). According to these criteria, there were 3,099 heterozygous genetic variants in the exome sequencing data and 278,304 heterozygous SNPs in the hapmap2 imputed data. We merged these into a final set of 279,885 unique candidate heterozygous genetic variants in our dataset, at which we estimated MeDIP-seq read coverage using maq pileup.

We considered variants where MeDIP-seq coverage depth surpassed 8 reads and used the allele frequency of the reference allele (calculated in VarAllele) as a measure of ASM; excluding all variants that were not bi-allelic. We called a site within an individual as ASM only if the reference allele frequency was < 0.25 or > 0.75. Altogether, ASM calls were obtained for 17,261 variants. We excluded all SNPs in genomic regions reported to be problematic in alignment according to reference 58.

Blood-Brain comparison

We used MeDIP-seq levels from blood and multiple brain regions in two female donors from Davies et al. [37]. The brain regions included inferior frontal gyrus, middle frontal gyrus, left frontal gyrus, entorhinal cortex, superior temporal gyrus of the temporal cortex, visual cortex, and cerebellum. To assess tissue-specificity of methylation effects at the DMRs we first considered regions to have tissue-shared methylation if the methylation difference between blood and one brain region was less than 10% of the overall blood methylation level in each of the two individuals profiled. Five of the 9 significant MAP-DMRs (and 63%

of 100 top-ranked MAP-DMRs) met these criteria, including: MTMR12, chr4:165,977,000-165,978,000bp, MICAL2, ST6GALNAC3, and NFU1. Under more stringent criteria, if the mean difference between blood and all brain region was less than 10% of the overall blood methylation level, one MAP-DMR on chromosome 4 (and 8% of 100 top-ranked MAP-DMRs) had tissue-shared methylation levels. We also computed the correlation between brain and blood methylation levels, by using the mean methylation levels across both individuals in all brain regions and also in blood.

Genome Annotation and Gene Ontology

Gene annotations and DNA sequence features information were obtained from the UCSC genome browser. CpG Islands track information 63 was obtained from UCSC. Each methylation region was assigned to the gene with the nearest TSS from Refseq, and restricted analyses to genes that were within 50kb and up to 100kb of the TSS.

Histone modification ChIP-seq data were obtained from the Encode project from the CEPH HapMap LCL (GM12878) in the UCSC genome browser. We obtained data for genome-wide distribution of reads from ChIP-seq histone modifications for 7 modifications: H3K9ac, H3K27ac, H3K27me3, H3K4me1, H3K4me2, H3K4me3, H4K20me1. Each histone genome-wide distribution of reads was smoothed using a bandwidth of 100 and peaks were assigned using a stringent threshold of 1.0 as previously described in reference 64 .

Gene ontology analysis was performed using GOrilla to report significant (P = 0.001) term enrichment. The results highlighted enrichment of 3 processes: iron-sulfur cluster assembly (GO:0016226), metallo-sulfur cluster assembly (GO:0031163), and vasoconstriction (GO:0042310).

Supplementary References

[62] Andrew, T., Hart, D. J., Snieder, H., de Lange, M., Spector, T. D., and MacGregor, A. J. Twin Res 4(6), 464–7 Dec (2001).

[63] Gardiner-Garden, M. and Frommer, M. J Mol Biol 196(2), 261–82 Jul (1987).

[64] Bell, J. T., Pai, A. A., Pickrell, J. K., Gaffney, D. J., Pique-Regi, R., Degner, J. F., Gilad, Y., and Pritchard, J. K. Genome Biol 12(1), R10 (2011).
